# Increased miR-3074-5p expression promotes M1 polarization and pyroptosis of macrophages via ERα/NLRP3 pathway and induces adverse pregnancy outcomes in mice

**DOI:** 10.1038/s41420-024-01941-4

**Published:** 2024-04-10

**Authors:** Long Yang, Hao-Ran Xu, Xuan Zhang, Yan Shi, Jia-Xin Shi, Qian-Qian Chen, Xiao-Rong Shen, Ya-Ping He, Jia-Nan Tang, Wen-Wen Gu, Jian Wang

**Affiliations:** 1 NHC Key Laboratory of Reproduction Regulation, Shanghai Key Lab of Health and Diease Genomics, Shanghai Institute for Biomedical and Pharmaceutical Technologies, School of Pharmacy, Fudan University, Shanghai, 200237 China; 2 Reproductive Medicine Center, Key Laboratory of Clinical Laboratory Diagnosis and Translational Research of Zhejiang Province, The First Affiliated Hospital of Wenzhou Medical University, Wenzhou, Zhejiang 325000 China

**Keywords:** Inflammasome, Reproductive disorders, Diagnostic markers, Mechanisms of disease, Cell death and immune response

## Abstract

Decidual macrophages (dMϕs) play critical roles in regulation of immune-microhomeostasis at maternal-fetal interface during pregnancy, but the underlying molecular mechanisms are still unclear. In this study, it was found that litter size and fetal weight were significantly reduced, whereas the rate of embryo resorption was increased in miR-3074-5p knock-in (3074-KI) pregnant mice, compared to that of wild-type (WT) pregnant mice. Plasma levels of pro-inflammatory cytokines in 3074-KI pregnant mice were also significantly elevated compared to WT pregnant mice at GD7.5. The quantity of M1-Mϕs in uterine tissues of 3074-KI pregnant mice was significantly increased compared to WT pregnant mice at GD13.5. Estrogen receptor-α (ERα) was validated to be a target of miR-3074-5p. Either miR-3074-5p overexpression or ERα knockdown promoted transcriptional activity of NF-κB/p65, induced M1*-*polarization and pyroptosis of THP1-derived Mϕs, accompanied with increased intracellular levels of cleaved Caspase-1, cleaved IL-1β, NLRP3, cleaved GSDMD and ASC aggregation. Furthermore, ERα could not only bind to NLRP3 or ASC directly, but also inhibit the interaction between NLRP3 and ASC. The endometrial miR-3074-5p expression level at the middle secretory stage of repeated implantation failure (RIF) patients was significantly decreased compared to that of control fertile women. These data indicated that miR-3074-5p could promote M1 polarization and pyroptosis of Mϕs via activation of NLRP3 inflammasome by targeting ERα, and the dysregulation of miR-3074-5p expression in dMϕs might damage the embryo implantation and placentation by interfering with inflammatory microenvironment at the maternal-fetal interface during early pregnancy.

## Introduction

Appropriate inflammatory microenvironment at the maternal-fetal interface plays an important role in successful establishment and maintenance of pregnancy. Pregnancy period can be commonly divided into three of inflammatory phases, including implantation (inflammatory), active gestation (anti-inflammatory), and parturition (inflammatory) [1, 2]. Disruption of the delicate inflammatory dynamics at the maternal-fetal interface could lead to various adverse pregnancy outcomes. Decidual macrophages (dMϕs) are the second most abundant decidual immune cell type [3–5] and involved in the immune tolerance, trophoblast invasion, tissue and vascular remodeling, embryo growth and initiation of parturition at the maternal-fetal interface during pregnancy [6, 7]. Generally, Mϕs are classified as M1 and M2 subtypes based on the different functions and cytokine production [8]. Both the quantity and proportion of M1 and M2 dMϕs are altered to compose the inflammatory dynamics at the maternal-fetal interface [4]. Thus, abnormal death or polarization of dMϕs in early pregnancy can lead to recurrent miscarriages (RM) [9, 10], preterm labor (PTL) [11], preeclampsia (PE) [12], and fetal intrauterine growth restriction (IUGR) [13].

Inflammasomes are multiprotein signaling platforms that control the inflammatory response and coordinate antimicrobial host defences, including those that take place during pregnancy. NLRP3, the most well-characterized inflammasome sensor molecule, is a tripartite protein of the NLR family and associated with a diverse range of diseases [14]. Upon stimulation, components of the NLRP3 inflammasome are recruited and assembled leading to cleavage of pro-Caspase1 into its active form, which in turn cleaves pro-IL-1β and pro-IL-18 into their mature forms and induce pyroptosis (inflammatory cell death) [15, 16]. Accumulating evidence suggests that in the placenta, the activation of the NLRP3 inflammasome is involved in the pathogenesis of pregnancy syndromes associated with placental inflammation [17, 18]. However, the role of NLRP3 played in dMϕs is still unclear.

It was observed in our previous studies that, miR-3074-5p expression levels in placental villus and decidual tissues of RM patients were significantly upregulated [19, 20]; and increased miR-3074-5p expression showed inhibitory effects on invasion of human extravillous trophoblast (EVT) cells [21] and decidualization of human endometrial stromal cells (ESCs) in vitro. And it was observed that miR-3074-5p was widely expressed in various cells of decidual tissues [20]. However, whether or not miR-3074-5p participates in regulating activities of decidual immune cells, and the increased miR-3074-5p expression might lead to the pregnancy loss in vivo, are still unclear. As the research interests of our lab have been focused on the dMϕs polarization [22, 23], and we found that estrogen receptor α (ERα), of which was reported to be involved in regulation of NLRP3 inflammasome activities [24], was a predict target of miR-3074-5p, thus, in the present study, a miR-3074-5p-knock-in mice model, as well as the in vitro induced M1 polarization models of the human peripheral blood monocyte cell line THP1-derived Mϕs (THP1-Mϕs) and primary mouse bone marrow-derived Mϕs (BMDMs), were established and applied to observe the effect of miR-3074-5p over-expression on pregnancy outcomes in mice, and to investigate roles of miR-3074-5p/ERα/NLRP3 pathway played in M1 polarization and pyroptosis of Mϕs in vitro.

## Results

### MiR-3074-5p over-expression led to adverse pregnancy outcomes and enhanced inflammatory response in pregnant mice

**Fig. 1:**
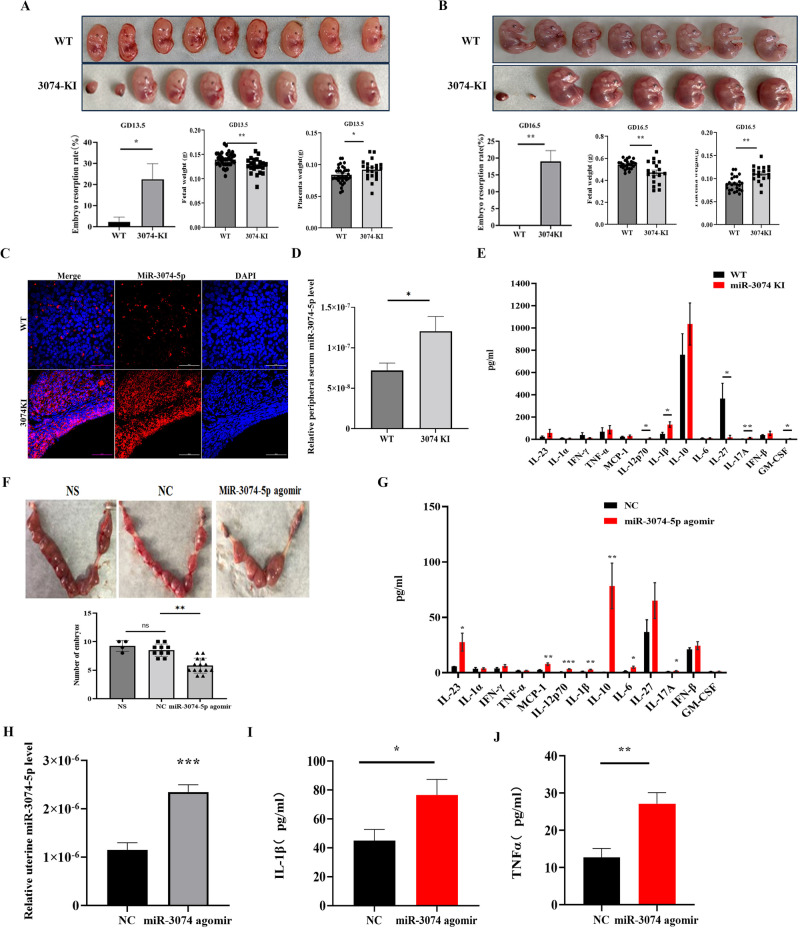
Overexpression of miR-3074-5p led to adverse pregnancy outcomes in mice. **A** Upper: representative images of fetuses respectively collected from a wild type (WT) pregnant and a miR-3074-5p-knock-in (3074-KI) pregnant mouse at GD13.5 (vaginal plug was defined as GD0.5); below: the embryo resorption rate, fetal weight and placenta weight in GD13.5 pregnant WT (*n* = 4) and 3074-KI (*n* = 3) mice. **B** Upper: representative images of fetuses respectively collected from a WT mouse and a 3074-KI mouse at GD16.5; below: the embryo resorption rate, fetal weight and placenta weight in GD16.5 pregnant WT (*n* = 3) and 3074-KI (*n* = 3) mice. **C** Uterine miR-3074-5p expression in pregnant WT (*n* = 3) and 3074-KI (*n* = 3) mice at GD7.5 detected by in situ hybridization (miR-3074-5p, red; nuclei, blue). **D** Serum miR-3074-5p level in GD7.5 WT (*n* = 5) and 3074-KI (*n* = 5) mice detected by qPCR. Experiments were independently repeated three times. **E** Serum levels of cytokines in GD7.5 WT (*n* = 4) and 3074-KI (*n* = 4) pregnant mice determined by the multiplex cytokine arrays. Replicate wells were set for each sample. **F** Upper: representative images of uterine tissues collected respectively from pregnant mice at GD13.5; below: number of implanted embryos in GD13.5 pregnant mice; NS: pregnant mice were injected with saline at GD3.5 (*n* = 4), NC: pregnant mice were injected with negative control sequence at GD3.5 (*n* = 10), miR-3074-5p agomir: pregnant mice were injected with miR-3074-5p agomir at GD3.5 (*n* = 14). **G** Serum levels of cytokines in GD7.5 pregnant mice treated with negative control (NC) (*n* = 3) or miR-3074-5p agomir (miR-3074 agomir) (*n* = 3) determined by the multiplex cytokine arrays. Two replicate wells were set for each sample. **H** Uterine miR-3074-5p expression level of GD7.5 pregnant mice treated with NC (*n* = 3) or miR-3074-5p agomir (*n* = 3) detected by qPCR. Experiments were independently repeated three times. Uterine levels of IL-1β (**I**) and TNF-α (**J**) in GD7.5 pregnant mice treated with NC (*n* = 3) or miR-3074-5p agomir (*n* = 3) detected by ELISA. Three replicate wells were set for each sample. All data are presented as means ± SEM, differences were identified by unpaired *t* test, *p < 0.05, **p < 0.01,***p < 0.001, ns: *p* > 0.05.

Given it was previously observed that, miR-3074-5p expression levels in villus and decidual tissues of recurrent miscarriage (RM) patients were significantly elevated, the miR-3074-5p-knockin (3074-KI) mouse model was established here to reveal the effect of over-expressed miR-3074-5p on pregnancy outcomes in vivo. 3074-KI mice were obtained by mating miR-3074-5p^loxp/loxp^ mice with EIIA-cre mice, and the genotype of offsprings was identified (Supplementary Fig. S1A). It was found that, the litter size of 3074-KI pregnant mice was significantly reduced compared to that of wild-type (WT) pregnant mice (Supplementary Fig. S1B). Subsequently, the pregnancy outcomes were respectively observed on days 13.5 (GD13.5) and 16.5 (GD16.5) of pregnancy (the day of vaginal plug check was defined as GD0.5). The results showed that, the embryo weight of 3074-KI pregnant mice was significantly reduced, whereas the rate of embryo resorption, as well as the placenta weight, were obviously increased compared to that of WT pregnant mice (Fig. 1A, B), indicating that the reduced litter size of 3074-KI pregnant mice might be caused by pregnancy loss or/and abnormal placentation.

The uterine miR-3074-5p expression in adult female mice was detected by in situ hybridization, and it was observed that the miR-3074-5p signals (red) in uterine tissues of 3074-KI mice at GD7.5 was more abundant than that of WT mice (Fig. 1C). The peripheral serum miR-3074-5p level of the pregnant mice (GD7.5) was also detected by qPCR, and the results showed that the serum level of miR-3074-5p in 3074-KI mice was significantly higher than that of WT mice (Fig. 1D). It has been well understood that embryo implantation requires an active Th1 inflammatory response, while a Th2-humoral inflammation is required for pregnancy maintenance. Thus, we firstly observed the in vivo effects of miR-3074-5p over-expression on inflammatory response during the embryo implantation period by using the established 3074-KI mouse model. The peripheral levels of inflammatory factors in GD7.5 pregnant mice were determined by LEGEND plex Mouse Cytokine Panel (13-plex), and the results showed that, the serum levels of IL-12p70, IL-1β, IL-27, IL-17A and GM-CSF in GD7.5 3074-KI mice (miR-3074 KI) were significantly elevated compared to that in GD7.5 WT mice (WT) (Fig. 1E).

MiRNA agomir is a specially chemically modified miRNA agonist that acts by simulating endogenous miRNA entry into the miRISC complex to regulate the expression of target gene mRNA. To furtherly authenticate the adverse effect of up-regulated uterine miR-3074-5p expression on pregnancy outcomes in mice, GD3.5 (day of implantation) WT pregnant mice were treated by intrauterine injection of miR-3074-5p agomir, and it was found that the quantity of implanted embryos in GD13.5 miR-3074-5p agomir-treated pregnant mice was significantly reduced compared to that of negative control-treated (NC) pregnant mice (Fig. 1F). Likewise, the serum levels of IL-23, MCP-1, IL-12p70, IL-1β, IL-10, IL-6 and IL-17A in GD7.5 pregnant mice treated with miR-3074-5p agomir at GD3.5 were obviously elevated compared to that in GD7.5 pregnant mice treated with NC at GD3.5 (Fig. 1G). In addition, uterine miR-3074-5p level, as well as IL-1β and TNF-α levels in uterine lysate, were determined by qPCR or ELISA in GD7.5 pregnant mice. It was found that, uterine levels of miR-3074-5p (Fig. 1H), IL-1β (Fig. 1I) and TNF-α (Fig. 1J) in miR-3074-5p agomir-treated pregnant mice were significantly increased compared to that in NC-treated pregnant mice, indicating a stimulating effect of miR-3074-5p on expression of systemic and local pro-inflammatory factors in the uterus during embryo implantation period in mice.

### MiR-3074-5p over-expression promoted M1-polarization of human macrophages by targeting ERα

**Fig. 2:**
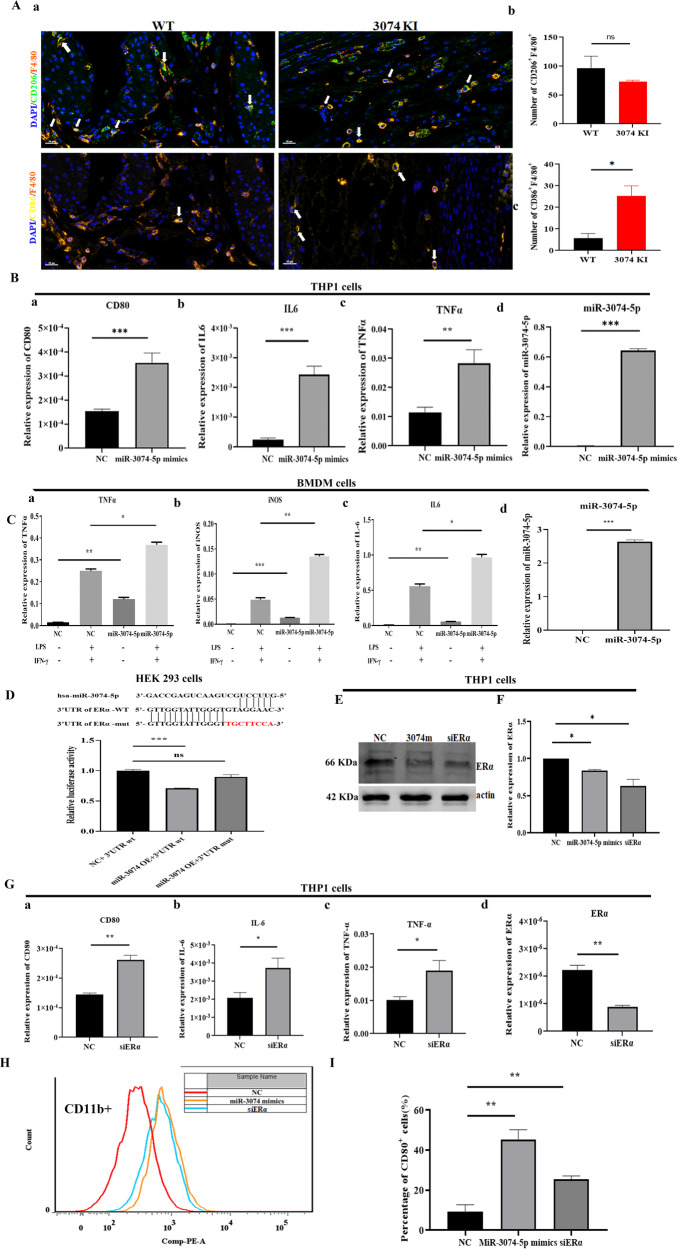
MiR-3074-5p induced M1-polarization of macrophages by targeting ERα. **A** (a) The representative images of multiplex immunohistochemistry staining of uterine tissue collected from a wild type pregnant mouse (WT) and a miR-3074-5p-knockin pregnant mouse (3074KI) at GD13.5; M1-subtype (F4/80^+^/CD86^+^) and M2-subtype (F4/80^+^/CD206^+^) macrophages (Mϕs) were stained in the paraffin sections, Orange: F4/80^+^, green: CD206^+^, yellow: CD86^+^; Scale bar = 20 μm; (b) the positive cell number of M1-subtype (F4/80^+^/CD86^+^) and M2-subtype (F4/80^+^/CD206^+^) Mϕs in 3074KI pregnant mice (*n* = 3) and WT pregnant mice (*n* = 3) at GD13.5. **B** Expression levels of CD80 (a), IL-6 (b), TNF-α (c) and miR-3074-5p (d) in THP1-derived Mϕs after 24 h of treatment with PMA (200 nM). Cells were transfected with negative control fragment (NC) or miR-3074-5p mimics (miR-3074-5p mimics) for 24 h, and then, mRNA levels of human M1-Mϕ markers (CD80, IL-6 and TNF-α) and miR-3074-5p were determined by RT-qPCR assay (*n* = 3 × 3). **C** Expression levels of TNF-α (a), iNOS (b), IL-6 (c) and miR-3074-5p (d) in Bone marrow-derived macrophages (BMDMs). Cells were transfected with NC or miR-3074-5p mimics for 24 h, and then, treated with LPS and IFNγ for another 24 h, mRNA expression levels of mouse M1-Mϕ markers (TNF-α, iNOS and IL-6) and miR-3074-5p were determined by RT-qPCR assay (*n* = 3 × 3). **D** Upper: Schematic representation of ERα 3ʹUTR sequence demonstrating predicted putative and mutated miR-3074-5p binding site (mutations in red); below: Relative luciferase activity in HEK293 cells co-transfected by negative control plasmid and wild type of ERα-3ʹUTR sequence (NC+3ʹUTRwt), or co-transfected by the miR-3074-5p overexpression plasmid and wild type ERα-3ʹUTR sequence (miR-3074OE+3ʹUTRwt), or co-transfected by the miR-3074-5p overexpression plasmid and mutant 3ʹ-UTR sequence of ERα (miR-3074OE+3ʹUTRmut) respectively. **E** Representative images of Western blotting detection of ERα protein expression levels in THP1 cells. Cells were respectively transfected by NC fragment (NC), miR-3074-5p mimics (3074 m), or ERα specific-siRNA (siERα). **F** Relative expression level of ERα protein in THP1 cells. Cells were respectively transfected by NC fragment (NC), miR-3074-5p mimics, or ERα specific-siRNA (siERα) for 48 h, and then, ERα expression level was detected by Western blot assay (*n* = 3). **G** Expression levels of CD80 (a), IL-6 (b) and TNF-α(c) and ERα (d) in THP1 cells. Cells were respectively transfected with negative control fragment (NC) or ERα-siRNA (siERα) for 24 h, and then, mRNA levels of M1 polarization markers (CD80, IL-6 and TNF-α) and ERα were determined by RT-qPCR (*n* = 3 × 3). **H** Flow cytometry assay of CD11b-FITC and CD80-PE on cell surface of THP1 cell. **I** The percentage of CD80-positive cells in the CD11b-positive cell population of THP1 cells transfected respectively by NC, miR-3074-5p mimics or siERα (*n* = 3 × 3). All data are shown as the mean ± SEM, *p < 0.05, **p < 0.01, ***p < 0.001, ns: *p* > 0.05.

In order to evaluate whether or not the increased inflammatory response and adverse pregnancy outcomes of 3074-KI pregnant mice were associated with the dysfunction of decidual macrophages (dMϕs), the effects of miR-3074-5p over-expression on Mϕs were investigated. Firstly, we examined the proportions of M2-dMϕs in uterine tissues collected from WT pregnant mice respectively at GD7.5 (late stage of pregnancy establishment) and GD13.5 (stage of pregnancy maintenance). As expected, it was found that, the relative number of M2-dMϕs (F4/80^+^/CD206^+^) at GD7.5 (3.3 ± 5.7) was significantly lower than that of GD13.5 (96.7 ± 35.7) (Supplementary Fig. S2). Subsequently, the effect of miR-3074-5p over-expression on polarization of dMϕs in pregnant mice at GD13.5 was determined. The results showed that, the relative number of M1-dMϕs (F4/80^+^/CD86^+^) in 3074-KI pregnant mice was significantly higher than that in WT pregnant mice. However, there was no significant difference in the number of M2-dMϕs (F4/80^+^/CD206^+^) between the two groups (Fig. 2A), indicating a stimulating effect of miR-3074-5p on M1 polarization of dMϕs during the period of pregnancy maintenance.

Then, the in vitro effect of over-expressed miR-3074-5p on Mϕs polarization was investigated by using the human peripheral blood monocyte cell line THP1. According to the well-established method [25], human Mϕs were induced from THP1 cells by treatment with PMA. Then, miR-3074-5p expression was up-regulated by transfection with miR-3074-5p mimics, while NC-transfected cells were used as control. The expression levels of human M1-Mϕs markers CD80, IL-6 and TNF-α were detected to evaluate the effect of over-expressed miR-3074-5p on M1 polarization of THP1-derived Mϕs. The results showed that, miR-3074-5p expression level, as well as expression levels of CD80, IL-6, and TNF-α, in miR-3074-5p mimics-tranfected THP1-derived Mϕs (miR-3074-5p mimics) were significantly increased compared to that of NC-transfected cells (NC) (Fig. 2B), suggesting a stimulating effect of miR-3074-5p on M1 polarization of human Mϕs. Meanwhile, primary mouse bone marrow-derived macrophages (BMDMs) were isolated and treated with LPS (100 ng/ml) and IFN-γ (20 ng/ml) to generate mouse M1-Mϕs in vitro as described [26].While the miR-3074-5p expression level was significantly up-regulated by transfection with miR-3074-5p mimics, expression levels of mouse M1-Mϕs markers, TNF-α, IL-6 and iNOS, were simultaneously increased in BMDMs compared to that in NC-transfected BMDMs (Fig. 2C). These results indicate that over-expressed miR-3074-5p could promote the M1 polarization of mouse Mϕs.

Furthermore, ERα was here validated to be a direct target gene of miR-3074-5p by dual-luciferase-reporter assay system in HEK293 cells. The wild-type (pRL-TK-ERα-3ʹUTR -wt) or mutant (pRL-TK-ERα-3ʹUTR-mut) 3ʹUTR sequence of ERα mRNA was respectively co-transfected with either miR-3074-5p overexpression plasmid (miR-3074-OE) or NC into HEK293 cells. And the results showed that, luciferase activity was significantly inhibited in miR-3074-OE cells compared to that in NC cells, whereas such an inhibitory effect was evaporated by the mutant in 3ʹUTR sequence of ERα (Fig. 2D), indicating that miR-3074-5p could inhibit ERα expression by directly targeting 3’UTR sequence of ERα mRNA. Subsequently, the effect of overexpressed miR-3074-5p on the ERα expression level in THP1 cells was examined. The results of western blot analysis showed that, the ERα expression levels were significantly decreased both in miR-3074-5p mimics-transfected cells (miR-3074-5p mimics) or the ERα specific siRNA-transfected cells (siERα), compared to that in NC-transfected cells (NC) (Fig. 2F). Then, we furtherly examined the effect of down-regulated ERα expression on the M1-polarization activity of Mϕs. As expected, the decreased ERα expression was accompanied with significantly increased expression levels of CD80, IL-6 in ERα specific siRNA-transfected THP1-derived Mϕs (siERα), compared to that in NC-transfected Mϕs (NC)(Fig. 2G), indicating that the down-regulated ERα expression could promote the M1-polarization of human Mϕs. To verify these findings, the M1-Mϕs surface marker CD80 was measured by flow cytometry, and it was found that either miR-3074-5p overexpression or ERα knockdown could significantly induce the expression of CD80 on CD11b^+^ THP1 cells (Fig. 2I). Taken together, miR-3074-5p could induce the M1-polarization of Mϕs by targeting ERα.

### MiR-3074-5p over-expression activated NLRP3 inflammasome and provoked pyroptosis of macrophages

**Fig. 3:**
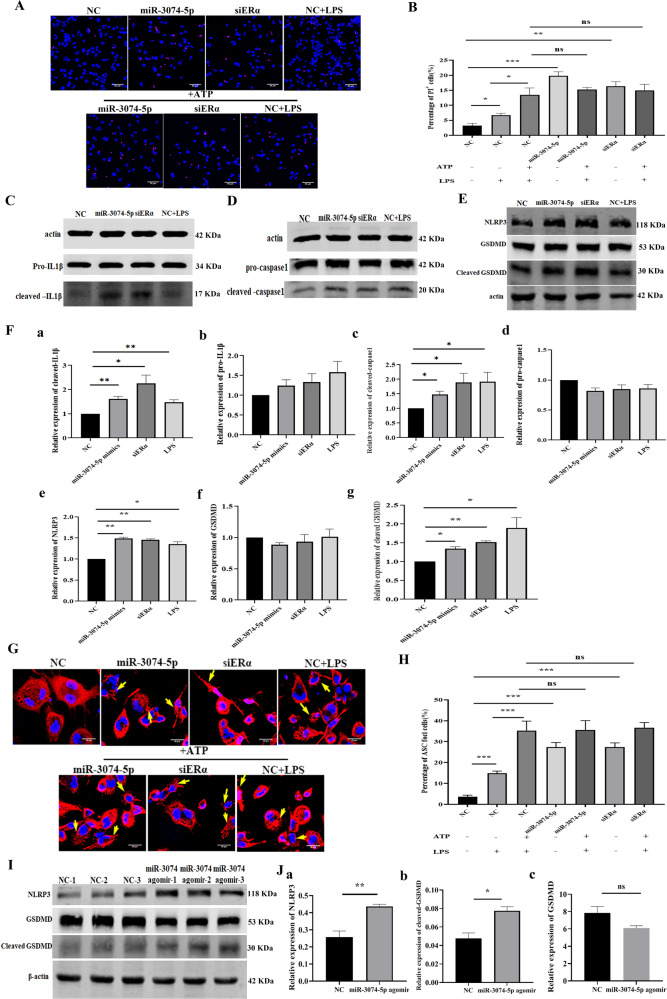
MiR-3074-5p promoted pyroptosis of THP1-derived Mϕs and uterine expression levels of pyroptosis-related proteins. **A** Representative images of propidium iodide (PI) staining.THP1-derived Mϕs were respectively transfected with negative control fragment (NC), miR-3074-5p mimics (miR-3074-5p) or ERα-siRNA (siERα), or treated with LPS (200 ng/ml) for 4 h and then treated with 5 mM ATP for 30 min to induce pyroptosis (red: PI, blue: hoechst33342, scale bar = 50 μm). **B** The percentage of PI-staining positive cells in total cells. PI-positive cells were counted on THP1-derived Mϕs from at least three independent fields per group. **C** Representative images of Western bolt assay of pro- and cleaved-IL1β proteins. **D** Representative images of Western bolt assay of pro- and cleaved-caspase1 proteins. **E** Representative images of Western bolt assay of NLRP3, GSDMD, and cleaved-GSDMD proteins. **F** The relative expression levels of cleaved-IL1β (a), pro-IL1β (b), cleaved-caspase1 (c), pro-caspase1 (d), NLRP3 (e), GSDMD (f), and cleaved-GSDMD (g) in THP1-derived Mϕs detected by Western blot assay. Cells were respectively transfected with NC fragment (NC), miR-3074-5p mimics (miR-3074-5p) or ERα-siRNA (siERα), or treated with LPS (200 ng/ml) (*n* = 3). **G** Representative images of immuno-fluorescence (IF) staining of ASC in THP1-derived Mϕs (red: Cy3-conjugated secondary antibody against ASC antibody, blue: hoechst33342, scale bar = 20 μm). **H** The percentage of THP1-derived Mϕs forming ASC aggregates. Cells were respectively transfected with negative control fragment (NC), miR-3074-5p mimics (miR-3074-5p) or ERα-siRNA (siERα), or treated with LPS (200 ng/ml) for 4 h and then treated with 5 mM ATP for 30 min to induce pyroptosis (*n* = 3 × 3). **I** Representative images of Western blot assay of uterine expression levels of NLRP3, GSDMD and cleaved-GSDMD protiens in GD8.5 pregnant mice. **J** Relative uterine expression levels of NLRP3 (a), GSDMD (b) and cleaved-GSDMD (c) protiens in mice detected by Western blot assy. Pregnant mice were respectively treated with NC fragment (*n* = 3) or miR-3074-5p agomir (*n* = 3), and the uterine tissue was collected at GD8.5. All data were presented as mean ± SEM. *p < 0.05, **p < 0.01, ***p < 0.001, ns: *p* > 0.05.

Given cell membrane damage was observed in THP1-derived Mϕs transfected with miR-3074-5p mimics (Supplementary Fig. S3), and the miR-3074-5p over-expression caused a significant increase in serum IL-1β level in mice, we speculated that miR-3074-5p might induce pyroptosis of Mϕs. Thus, firstly, we determined the cell membrane rupture of THP1 cells by Propidium Iodide (PI) staining, and the results showed that, significantly increased PI positive staining (red) were detected in miR-3074-5p over-expressed (miR-3074-5p) or ERα down-regulated (siERα) THP1 cells, indicating that miR-3074-5p might promote whereas ERα might inhibit pore formation and membrane rupture of THP1 cells. And this stimulating effect on THP1 cells pyroptosis was similar to that of caused by treatment with LPS plus ATP (Fig. 3B).

Subsequently, the expression levels of pyroptosis-related proteins were assessed by Western blot analysis. It was found that, the expression levels of cleaved IL-1β, cleaved Caspase-1, NLRP3, and cleaved GSDMD in THP1-derived Mϕs transfected with miR-3074-5p mimics or ERα-siRNA (siERα) were notably increased compared to that in THP1-derived Mϕs transfected with NC (Fig. 3F). Meanwhile, immunofluorescence staining results showed that, the percentage of ASC specks, which is required for Caspase-1 activation, was significantly increased in miR-3074-5p mimics or ERα-siRNA transfected Mϕs (Fig. 3H). Furthermore, uterine expression levels of pyroptosis related proteins were also determined by Western blot assay after intravenous injection of miR-3074-5p agomir or its NC fragment. The results showed that, the uterine expression levels of NLRP3 and cleaved GSDMD in miR-3074-5p agomir-treated mice were significantly increased compared to that in NC-treated mice (Fig. 3J). These results indicated that miR-3074-5p might provoke Mϕs pyroptosis through activation of NLRP3 inflammasome.

### MiR-3074-5p activated NLRP3 inflammasome by targeting ERα

**Fig. 4:**
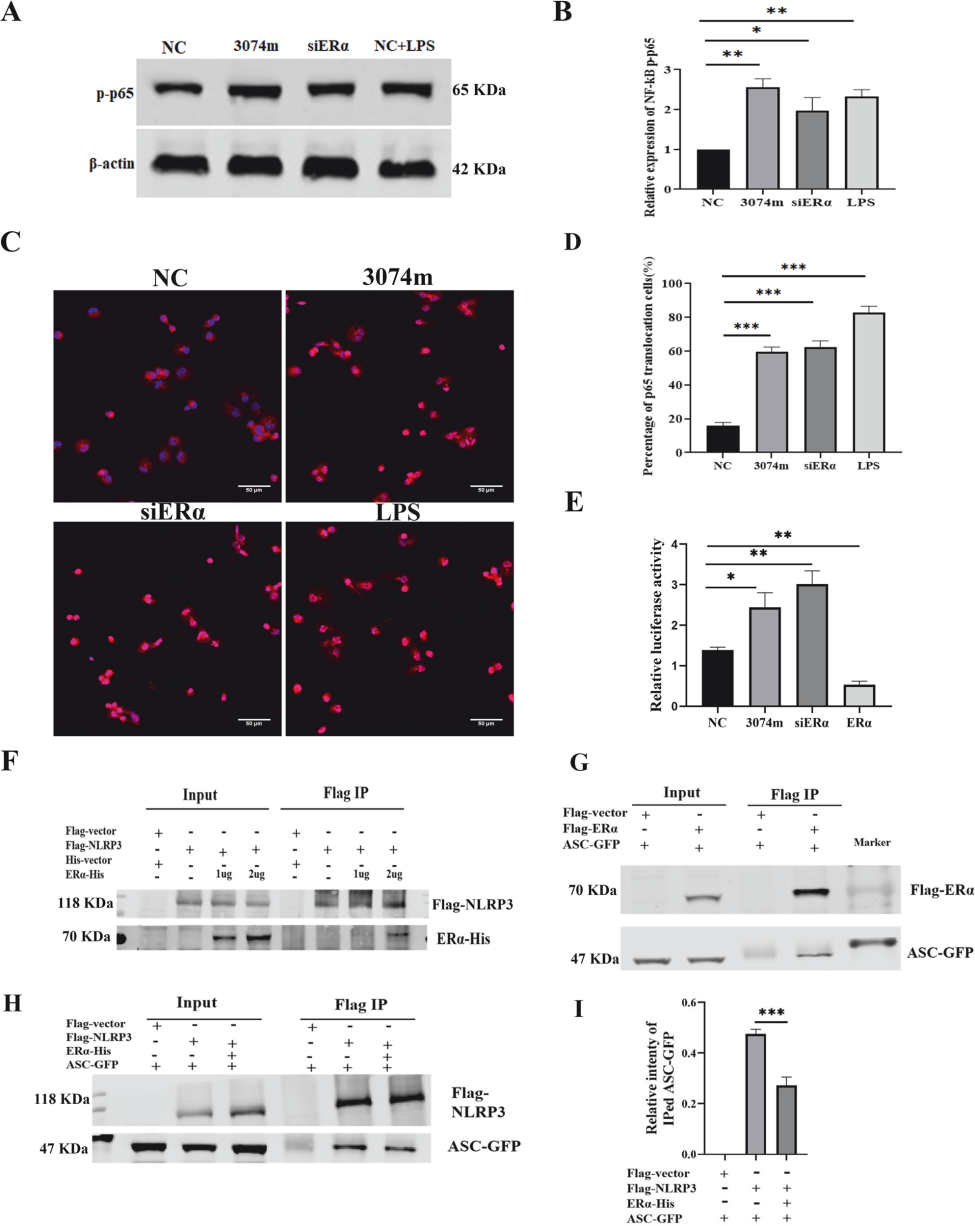
MiR-3074-5p activated NLRP3 inflammasome by targeting ERα in macrophages. **A** Rrepresentative images of Western blot assay on expression level of phosphorylated NFκB/p65(S276) protein in THP1-derived Mϕs. **B** Relative expression level of phosphorylatedNFκB/p65 (S276) protein in THP1-derived Mϕs detected by Western blot assay (*n* = 3). **C** Representative images of confocal immunofluorescence microscopy analysis of the NF-κB/p65 proteins translocated into the nuclei of THP1-derived Mϕs. **D** Percentage of cells with NFκB/p65 nuclei translocation (*n* ≈ 3 × 50 cells). **E** NFκB/p65 luciferase reporter activity relative to negative control in HEK293 cells following indicated treatments (*n* = 3). **F** HEK293T cells were co-transfected with different concentration of ERα-His plus Flag-NLRP3 or Flag vector for 24 h. Cell lysates were immunoprecipitated using anti-Flag beads and analyzed using anti-His and anti-Flag antibody. **G** HEK293T cells were co-transfected with ASC-GFP plus Flag-ERα or Flag vector for 24 h. Cell lysates were immunoprecipitated using anti-Flag beads and analyzed using anti-GFP and anti-Flag antibody. **H** HEK293T cells were co-transfected with ERα-His plus Flag-NLRP3 or Flag vectorand ASC-GFP for 24 h. Cell lysates were immunoprecipitated using anti-Flag beads and analyzed using anti-GFP and anti-Flag antibody. **I** The quantification data analysis of ASC-GFP immunoprecipitated by anti-Flag beads (*n* = 3). NC: cells were transfected with NC fragment; miR-3074-5p: cells were transfected with miR-3074-5p mimics; siERα: cells were transfected with ERα-siRNA; LPS: cells were treated by LPS (200 ng/ml); ERα: cells were transfected with ERα-expression recombinant plasmid. All data are shown as the mean ± SEM. *p < 0.05, **p < 0.01, ***p < 0.001.

Given ERα could suppress NF-κB activity and was validated to be a target of miR-3074-5p, we speculated that miR-3074-5p might induce activation of NF-κB pathway by reducing ERα expression. As expected, it was found that, phosphorylated NF-κB/p65 protein level was significantly increased in THP1 cells transfected with miR-3074-5p mimics or ERα-siRNA, as well as LPS stimulation, compared to that in NC-transfected cells (Fig. 4B). Then, immunofluorescence staining was performed and the results showed that either overexpression of miR-3074-5p or down-regulation of ERα promoted the nuclear translocation of NF-κB/p65 protein in THP1 cells (Fig. 4D). Furthermore, the dual-luciferase assay was carried out to determine whether miR-3074-5p directly regulate the activation of NF-κB, and it was observed that miR-3074-5p-overexpression or ERα-knockdown promoted transcriptional activity of NF-κB/p65 (Fig. 4E).

As the NLRP3 expression was also induced by miR-3074-5p overexpression or ERα knockdown (Fig. 3E), it could be reasonably suggested that miR-3074-5p might activate NLRP3 inflammasome through ERα/NF-κB signaling pathway. In addition, as ERα was reported to be able to directly activate NLRP3 inflammasome, we performed IP assay to evaluate the binding activity of ERα with NLRP3. The results showed that ERα could bind to NLRP3 through the PYD domain of NLRP3 (Fig. 4F and Supplementary Fig. S4). For NLRP3 recruits ASC through PYD domain to activate Caspase-1, we therefore performed IP assay to evaluate the binding of ERα with ASC, and it was found that ERα not only binds to ASC directly (Fig. 4G), but also inhibits the interaction between NLRP3 and ASC (Fig. 4H, I), indicating that ERα might inhibit NLRP3 inflammasome activation by suppressing NF-κB activity or/and disturbing the formation of NLRP3/ASC/Caspase1 complex, and miR-3074-5p could therefore activate NLRP3 inflammasome by targeting ERα.

### Conditional medium of miR-3074-5p over-expressed THP1-derived Mϕs inhibited the outgrowth of HTR-8/SVneo cell spheres

**Fig. 5:**
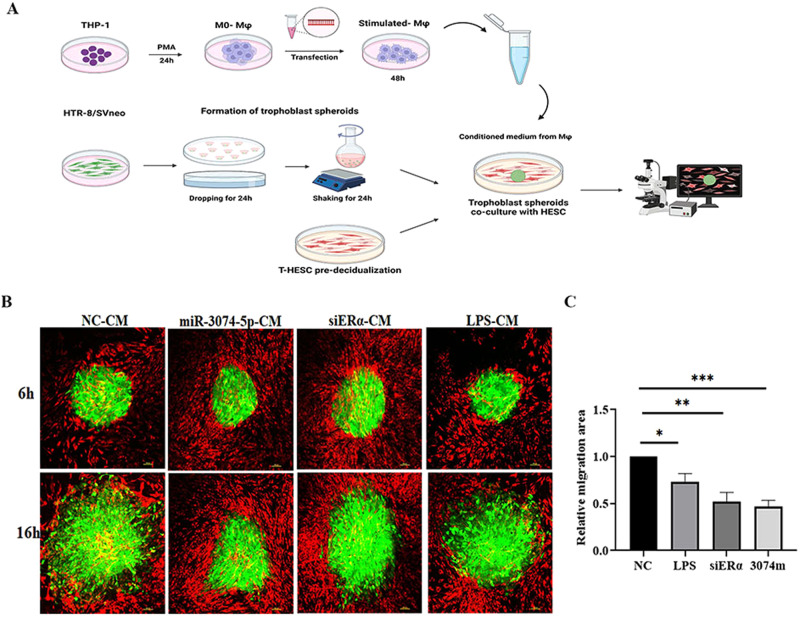
Conditional media from miR-3074-5p over-expressed macrophages inhibited outgrowth of extravillous trophoblasts spheres. **A** Schematic diagram of the co-culture model of human extravillous trophoblast cell line HTR-8/SVneo and decidualized human endometrial stromal cell line T-HESC. **B** Representative images of the HTR-8/SVneo cells co-cultured with T-HESCs respectively for 6 h and 16 h. HTR-8/SVneo cells were transfected with plvx-EGFP plasmid that stably expressed EGFP fluorescent protein (green), and cultured into cell spheroids before co-cultured with T-HESC. T-HESC cells were labeled by a lipophilic membrane dye PKH26 staining (red). **C** Trophoblast spheroids were co-cultured with T-HESC cells for 6 h, then 50% of the conditional media (CM) of THP1-derived Mϕs transfected respectively with negative control (NC), miR-3074-5p mimics (3074 m), ERα-siRNA (siERα) for 48 h, or treated with LPS (200 ng/ml) for 24 h, was added to the culture media of HTR-8/SVneo cells. HTR-8/SVneo cells were continuously co-cultured with T-HESC cells for 10 h, and real-time images were taken at 1-h intervals by confocal microscopy. The histogram shows the relative migration area of trophoblast cells calculated. Data are shown as the mean ± SEM of at least three independent experiments. *p < 0.05, **p < 0.01, ***p < 0.001.

To demonstrate whether or not the miR-3074-5p over-expression in dMϕs might affect the activities of extravillous trophoblasts (EVTs) via paracrine manner at the maternal-fetal interface, we established a time-lapse confocal imaging model of the human EVT cell line HTR-8/SVneo spheroids co-cultured with the human endometrial stromal cell line T-HESCs in vitro. THP1-derived Mϕs were transfected respectively with negative control fragment (NC), miR-3074-5p mimics or ERα-siRNA, or treated with LPS, and the four supernatant samples were collected 48 h after transfection as conditional medium (CM). These collected CM samples of Mϕs were added to the culture medium of HTR-8/SVneo cells, and outgrowth of HTR-8/SVneo cell spheroids (green) on decidualized T-HESC cells (red) were assessed by real-time imaging of live cells for 10 h (Fig. 5A). The outgrowth area of HTR-8/SVneo cell spheroids was calculated, and it was found that, outgrowth of HTR-8/SVneo cell spheroids were significantly inhibited by the treatment with miR-3074-5p-CM (3074 m), siERα-CM (siERα) or LPS-CM (LPS), compared to that treated with NC-CM (NC) (Fig. 5C), suggesting that the over-expression of miR-3074-5p in dMϕs might interfere with activities of EVTs at the maternal-fetal interface. The representative time-lpase movies were presented as the supplementary data (Supplementary movie 1–4).

### Endometrial miR-3074-5p expression was decreased in repeated implantation failure (RIF) patients at the middle secretory stage

**Fig. 6:**
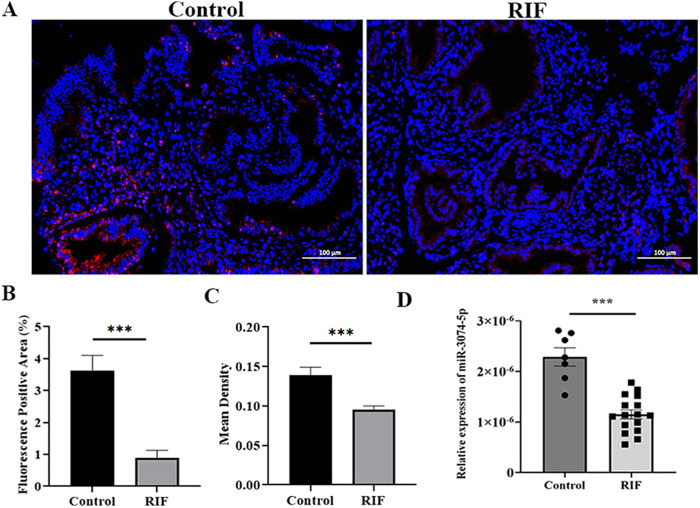
Endometrial miR-3074-5p expression at the mid-secretory phase was significantly decreased in repeated implantation failure (RIF) patients. **A** Representative images of in situ hybridization (miR-3074-5p, red; nuclei, blue). **B** Statistical results of fluorescence miR-3074-5p signals positive area. **C** Mean density of endometrial tissue by fluorescence in situ hybridization. **D** Endometrial miR-3074-5p expression level detected by RT-qPCR. Fluorescence positive area = positive area/tissue area; Mean density = cumulative optical density/positive pixel area; RIF repeated implantation failure patients (*n* = 17); Control: healthy women of childbearing age (*n* = 7). Data are shown as the mean ± SEM, ***p < 0.001.

For an appropriate inflammatory microenvironment is critical for embryo implantation, and miR-3074-5p showed stimulating effects on inflammatory response, we determined the association of endometrial miR-3074-5p expression level with RIF to initially explore the potential role of miR-3074-5p played in the pathogenesis of RIF. Endometrial tissues were collected from RIF patients (*n* = 16) and healthy control women (*n* = 7) at the middle secretory stage. In situ hybridization in paraffin-embedded specimen was performed to analyze the expression and location of miR-3074-5p in human endometrial tissues. The fluorescent positive area and fluorescent intensity of miR-3074-5p signals were measured. The results showed that the abundance of miR-3074-5p signals (red) were obviously reduced in endometrial tissues from RIF patients compared to that from control women (Fig. 6A–C). We further verified these results by RT-qPCR using endometrial samples from RIF patients and controls, and miR-3074-5p expression level was shown to be significantly decreased in RIF patients compared to control women (Fig. 6D), indicating that abnormally reduced endometrial miR-3074-5p expression might lead to the impaired endometrial receptivity by interfering with the establishment of a pro-inflammatory microenvironment.

## Discussion

In this study, we found that up-regulated miR-3074-5p expression led to embryo loss and abnormal fetus development in mice, accompanied with not only the elevated serum levels of pro-inflammatory cytokines at the final stage of implantation and initial stage of placentation, but also the increased quantity of M1-dMϕs at the final stage of placentation. MiR-3074-5p over-expression showed stimulating effects on M1 polarization and pyroptosis, as well as the production of pro-inflammatory cytokines in Mϕs. ERα was validated to be a target of miR-3074-5p, and both up-regulated miR-3074-5p expression and down-regulated ERα expression promoted the activation of NLRP3 inflammasome in Mϕs. Furthermore, conditional media of miR-3074-5p over-expressed Mϕs presented an inhibitory effect on outgrowth and invasion of HTR-8/SVneo cells on decidualized T-HESCs. And a reduced endometrial miR-3074-5p expression was observed in RIF patients at the middle secretory stage.

Although the significantly increased miR-3074-5p expression was previously observed both in villus and decidual tissues of RM patients [20, 21, 27], the relationship between the increased miR-3074-5p expression and the pregnancy loss has still not yet been explored. Being restricted by the medical ethics, we established a miR-3074-5p-knockin (3074-KI) mouse model here to investigate the in vivo effects of increased miR-3074-5p expression on pregnancy outcomes. The uterine miR-3074-5p over-expression of 3074-KI mice was confirmed by the significantly increased serum miR-3074-5p level and more abundant uterine miR-3074-5p signals detected in GD7.5 pregnant 3074-KI mice. And as expected, the reduced litter size and fetal weigh, as well as enhanced embryo absorption rate and placental weigh, were observed in 3074-KI pregnant mice, indicating miR-3074-5p over-expression could lead to pregnancy loss and abnormal fetal development.

Meanwhile, seeing that the established 3074-KI mouse model is not the uterine tissue specific knock-in model, the uterine specifically miR-3074-5p over-expression pregnant mouse model was parallelly established by intrauterine injection of miR-3074-5p agomir at the day 3.5 of pregnancy (GD3.5) before embryo enter into uterus. It was found that, uterine miR-3074-5p level, as well as uterine levels of IL-1β and TNF-α, in pregnant mice treated with miR-3074-5p agomir were excessively up-regulated at GD7.5, and the number of implanted embryos of miR-3074-5p agomir-treated GD13.5 pregnant mice was significantly reduced, indicating that increased uterine miR-3074-5p level might enhance inflammatory activities at the maternal-fetal interface and cause pregnancy loss in mice. Consistently, the peripheral serum levels of pro-inflammatory cytokines of 3074-KI pregnant mice at GD7.5 were detected to be significantly increased. Thus, it could be reasonably concluded that, uterine miR-3074-5p over-expression at early pregnancy might lead to adverse pregnancy outcomes at least partially through the enhancement of inflammation in mice.

Surprisingly, the serum levels of IL-10 and IL-6, two anti-inflammatory cytokines, were also significantly increased in mice treated with exogenous miR-3074-5p. IL-10 and IL-6, as pleiotropic regulators, can be secreted by a diverse set of cells in maternal-fetal interface, and the decreased IL-10 level was associated with adverse pregnancy outcomes, and exogenous administration of IL-10 could alleviate fetal loss induced by LPS [28]. Interestingly, it was reported that pro-inflammatory stimuli such as LPS could also induce the production of IL-10 and IL-6 [29, 30]. Thus, we speculated that miR-3074-5p agomir might also lead to the feed-back elevation of anti-inflammatory factors. However, this feed-back increase in anti-inflammatory factors is not enough to mitigate the damage caused by pro-inflammatory injury, ultimately leading to embryo loss. At this time, if further supplementation of exogenous anti-inflammatory factors reaches a certain level, the inflammatory damage caused by miR-3074-5p may be alleviated, but this needs to be confirmed by further studies.

A successful pregnancy requires precise immunological adaptations regulated by the maternal immune system to support fetal growth while simultaneously protecting mother and fetus against microbial challenges [31]. Monocytes in maternal circulation and tissue-resident macrophages at the maternal-fetal interface play a critical role in this delicate balance. During the period of pregnancy, macrophages (Mϕs) are abundantly present in the decidua, and distributed around extravillous trophoblasts (EVTs) [5, 32]. Mϕs have traditionally been defined as M1 (pro-inflammatory) or M2 (anti-inflammatory) subtype based on their cytokine production and function [33]. The shift of M1/M2 polarization of decidual Mϕs (dMϕs) is important for immunological adaptations at the maternal-fetal interfance [34], and aberrantly dMϕs polarization could lead to pregnancy complications, including RM, PE and preterm birth [4]. Interestingly, although no difference in quantity of M2-type dMϕs was observed in 3074-KI pregnant mice at GD13.5, the quantity of M1-type dMϕs in 3074-KI pregnant mice was detected to be significantly increased compared to WT pregnant mice at GD13.5, suggesting that miR-3074-5p over-expression might induce the M1 polarization dMϕs to enhance the inflammatory activities at the maternal-fetal interface during the pregnancy.

Encouragingly, the stimulating effect of miR-3074-5p over-expression on M1 polarization of Mϕs was observed both in human THP1-derived and mouse BMDM-derived Mϕs in vitro. Unexpectedly, the enhanced pyroptosis of THP1-derived Mϕs was also observed in miR-3074-5p over-expressed THP1-derived Mϕs. Given it has been reported that there is signaling pathways crosstalk in regulating Mϕs pyroptosis and the M1 polarization, and once activated, both secrete pro-inflammatory cytokines. Abnormal pyroptosis at the maternal-fetal interface has recently been highlighted in PE and RM [35, 36], we therefore paid attention on mechanisms underlying roles of miR-3074-5p played both in M1 polarization and pyroptosis of Mϕs. As the inhibition of ERα signaling activity could promote M1 polarization and pyroptosis of Mϕs [37, 38], the inhibitory effect of miR-3074-5p on ERα expression by directly targeting the 3ʹUTR sequence of ERα mRNA was validated here at the first time. And similar with the up-regulation of miR-3074-5p, the down-regulation of ERα can also promote M1 polarization and provoke pyroptosis of THP1-derived Mϕs, indicating that miR-3074-5p might induce M1 polarization and pyroptosis of Mϕs by targeting ERα. However, lack of the rescue experiment data that whether or not the ERα over-expression could effectively attenuate the effects of miR-3074-5p over-expression made this conclusion not confident enough. This should be addressed in our next investigation.

Classical cell pyroptosis is mediated by inflammasome, a kind of intracellular supramolecular complexes comprising a sensor molecule belong to NLR-containing or ALR protein families, the adaptor apoptosis­associated speck-like protein containing a CARD (ASC), and the effector protease caspase 1. Inflammasome assembly is accompanied by GSDMD cleavage and IL-1β release, and plays a central role in inflammatory-associated diseases [39]. Among recognized members of the NLR family, the cytosolic innate immune signaling receptor NLRP3 is most extensively investigated [40]. NLRP3 responds to highly diverse stimuli, including ATP, bacterial toxins, micro-crystalline substances, lipid particles, bacteria, and viruses. Once activated, NLRP3 nucleates the assembly of inflammasome and binds to ASC, successively leading to caspase 1-mediated proteolytic activation, GSDMD cleavage to form pores in the plasma membrane, cell swelling and membrane rupture, leakage of mature IL-1β, and finally pyroptotic cell death. In recent years, studies have confirmed the critical role of NLRP3 inflammasome in gynecological disorders and obstetrical complications. Targeting the activation of the NLRP3 inflammasome has become a potential therapeutic strategy [17, 41].

The NLRP3 inflammasome consists of a pyrindomain (PYD), a NACHT domain required for nucleotide binding with ATPase activity, and a leucine-rich repeat (LRR) domain [42]. Activation of NLRP3 inflammasome includes at least two-steps in most cell types, promotes NLRP3 expression and elicits inflammasome formation [43]. While promoting production of numerous inflammatory chemokines, cytokines, and cytokine precursors, including pro-IL1β, transcription factor nuclear factor κB (NFκB) also induce NLRP3 expression and is therefore important for inflammasome priming and assembly [44]. Since ERα represses NF-κB activity [45, 46], we firstly tested whether miR-3074-5p had effect on the activation of NF-κB. And it was found here that, either miR-3074-5p over-expression or ERα knockdown, as well as LPS stimulation, could induce phosphorylation and nuclear translocation of NF-κB/p65 protein. Furthermore, both miR-3074-5p over-expression and ERα knockdown led to increased expression of NLRP3 and mature IL-1β, membrane permeability and aggregation of ASC, as well as cleavage of Caspase1 and GSDMD, in THP1-derived Mϕs, indicating that miR-3074-5p might induce macrophage pyroptosis by activating NLRP3 inflammasome via ERα/NF-κB signaling pathway. In addition, it was found here at the first time that, ERα could directly bind to NLRP3 through the PYD domain, and not only bind to ASC directly, but inhibit the interaction of NLRP3 and ASC. Thus, miR-3074-5p might also induce macrophage pyroptosis without NF-κB by relieving the effects of ERα on interaction of NLRP3 and ASC.

Insufficient EVTs invasion is associated with pregnancy complications including RM, PE and fetal intrauterine growth restriction [47, 48], and various mediators secreted by dMϕs regulates EVTs activities in a paracrine manner [49–51]. M1-dMϕs exerted inhibitory effects on proliferation, apoptosis, invasion and migration of EVTs, whereas M2-dMϕs possessed the opposite effects [52, 53]. Thus, the effect of up-regulated miR-3074-5p expression in Mϕs on invasive ability of EVTs via the paracrine pathway was also determined by using a co-cultured EVTs/decidualized ESCs (decidual stromal cells, DSCs) model in vitro. And it was found that, the miR-3074-5p over-expressed Mϕs showed an inhibitory effect on outgrowth and invasion of EVTs on DSCs. For our previous study showed that up-regulated miR-3074-5p expression in EVTs could promote apoptosis but inhibit invasion of EVTs, we therefore speculated, abnormally increased miR-3074-5p expression at the maternal-fetal might lead to early pregnancy loss by inhibiting invasion of EVTs via direct effect of the miR-3074-5p over-expression in EVTs and indirect effect of its over-expression in dMϕs .

Nowadays, implantation failure after the embryo transfer has been a limited factor for the successful artificial human reproductive technique (ART). During the window of the implantation (WOI), endometrial Mϕs deflect to M1 activation and switch to a mixed M1/M2 profile to form an inflammatory environment to reach the receptive state of endometrium, when embryonic trophoblasts attach to the endometrial lining and invade the uterine stroma [34]. Given miR-3074-5p over-expression showed stimulating effects on M1 polarization of Mϕs and secretion of pro-inflammatory cytokines as stated above, we wonder whether or not the endometrial miR-3074-5p expression level is dysregulated during WOI period in implantation failure patients. Interestingly, endometrial miR-3074-5p expression level at the middle secretory stage (WOI period) was significantly reduced in the endometrial tissues collected from RIF patients, indicating that the decreased endometrial miR-3074-5p expression might lead to the impaired endometrial receptivity by inhibiting the M1 polarization of Mϕs and secretion of pro-inflammatory cytokines, and presenting a potential biomarker or target for clinical management of RIF patients. Thus, the dMϕs should be isolated from endometrial tissues of RIF patients at the middle secretory stage to determine the correction of miR-3074-5p expression level with M1/M2 profile, and peripheral blood levels of miR-3074-5p and its downstream factors should be examined to evaluate the potentiality for clinical application in our next investigation.

**Fig. 7:**
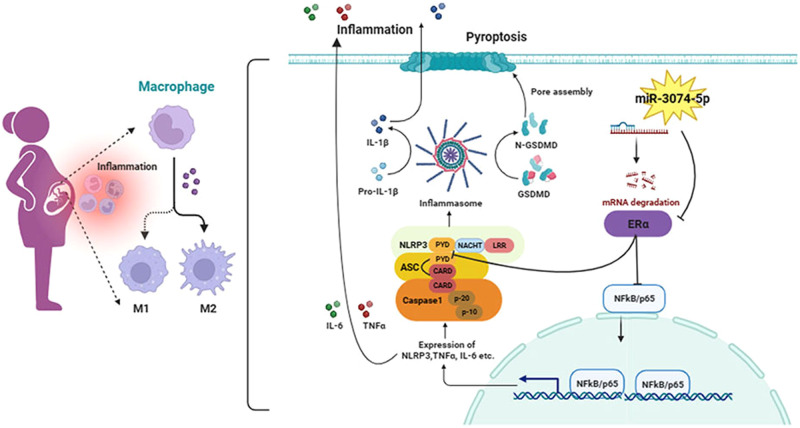
Schematic diagram of functional regulation of miR-3074-5p on M1 polarization and pyroptosis of decidual macrophages by targeting ERα, leading to adverse pregnancy outcomes. Abnormally increased miR-3074-5p expression in dMϕs at the early pregnancy could directly inhibit ERα expression, and subsequently activate NLRP3 inflammasome by activating NFκB signal pathway or/and promoting the interaction of NLRP3 and ASC, followed by the enhancement of M1 polarization and pyroptosis of dMϕs, disturbing the immune-microenvironment at the maternal-fetal interface and finally leading to embryo loss and abnormal fetal development.

In summary, this study indicated that, abnormally increased miR-3074-5p expression in dMϕs at the early pregnancy could directly inhibit ERα expression, and subsequently activate NLRP3 inflammasome by activating NFκB signal pathway or/and promoting the interaction of NLRP3 and ASC, followed by the enhancement of M1 polarization and pyroptosis of dMϕs (Fig. 7), disturbing the immune-microenvironment at the maternal-fetal interface and finally leading to embryo loss and abnormal fetal development. And peripheral or endometrial levels of miR-3074-5p and its related molecules present the potential biomarkers for clinical management of adverse pregnancy outcomes.

## Methods

### Cell lines and cell culture

The immortalized human monocytic cell line THP1 was purchased from ATCC (ATCC, Manassas, USA). THP1 cells were cultured in RPMI-1640 (Gibco, Thermo Fisher Scientific, United States) containing 10% heat-inactivated FBS (Gibco) and 0.055 mM 2-mercaptoethanol (Gibco). The human endometrial stromal cell line T-HESC was kindly provided by Prof. Hai-Bin Wang. The medium used for T-HESC cells was phenol red-free DMEM/F-12 (Gibco), supplemented with 10% charcoal-stripped fetal bovine serum (CS-FBS) (Biological Industries, Cromwell, CT), 3.1 g/L glucose (Sigma), 1 mM sodium pyruvate (Sigma), 1.5 g/L sodium bicarbonate (Sigma), 1% insulin-transferrin-selenium (ITS, Gibco), 500 ng/ mL puromycin (Sigma), and 50 U/mL penicillin–streptomycin (Gibco). HTR-8/SVneo was kindly provided by Prof. Yan-Ling Wang, and cultured in RPMI 1640 medium, supplemented with 10% FBS, 100 U/ml penicillin, and 100 μg/ml streptomycin (Gibco). Cells were incubated at 37 °C in a humidified atmosphere with 5% CO2. The cell lines were identified using STR analysis, and were routinely tested for mycoplasma contamination.

### Generation of miR-3074-5p knock-in mice and collection of tissue samples

EIIa-Cre transgenic mice were purchased from Cyagen Biosciences (Suzhou, China). MiR-3074-5p-KI mice were constructed as illustrated (Supplementary Fig. S5). To get miR-3074-5p knock-in (3074-KI) mice, EIIa-Cre^+/−^/miR-3074-5p^loxp/+^ mice (F1) were generated by crossing miR-3074-5p^loxp/loxp^ mice with EIIa-Cre transgenic mice (F0), and the F1 mice were subsequently crossed with miR-3074-5p^loxp/loxp^ mice to get F2 fetuses including Control (miR-3074-5p^loxp/+^) and 3074-KI (EIIa-Cre ^+/−^/miR-3074-5p^loxp/loxp^). After sacrificed by cervical dislocation respectively at GD7.5, GD13.5 or GD16.5, the fetuses and placentas were counted and weighed separately without blinding. And the collected uterine and placental tissues were fixed in 4% paraformaldehyde (PFA) (Sigma-Aldrich) and subjected to paraffin or frozen embedding. All experiments were carried out in accordance with standard laboratory animal care protocols that were approved by the Institutional Animal Care Committee of Shanghai Institute for Biomedical and Pharmaceutical Technologies (#2020-27). All mice were housed under a 12 h light/12 h dark cycle (light from 07:00 AM to 19:00 PM, temperature [23–25 °C] and relative humidity [40%–60%]), with adequate water and food supply. For the mating experiment, 8~10 week-old male and virgin female mice were cohoused from 5:00 PM to 8:00 AM, and the morning when vaginal plug was detected was recorded as the gestation day 0.5 of pregnancy (GD0.5).

### Treatment of pregnant mice with miR-3074-5p agomir

Adult wild type (WT) female and male C57BL/6 mice (6–8 weeks old) were purchased from SIPPR/BK Laboratory Animal Company (Shanghai, China). Adult female mice were mated with fertile males of the same strain to achieve pregnancy, and the day on which sperms were detected in the vaginal smear was defined as GD0.5 of pregnancy. The mice were randomly divided into two groups: For intravenous administration, pregnant mice were injected through the tail vein with 20 nM miR-3074-5p agomir (in 200μl normal saline) or 20 nM negative control fragment (in 200μl normal saline) (GenePharma Co., Shanghai, China) on GD3.5, and uterine tissue and peripheral blood samples were collected at GD7.5. For intrauterine injection, pregnant mice were randomly divided into three groups: injected through uterine horn respectively with 10 μl normal saline, 10 nM miR-3074-5p agomir (in 10 μl normal saline) or 10 nM negative control fragment (in 10 μl normal saline) (GenePharma Co., Shanghai, China) on GD3.5. Then, these treated pregnant mice were sacrificed by cervical dislocation at GD13.5 to count the number of embryos. All of the mice were caged at controlled temperature of 22-25 °C, and under a 12 h light: 12 h dark photoperiod. All the experiments were conducted in full compliance with standard laboratory animal care protocols approved by the Institutional Animal Care Committee of Shanghai Institute for Biomedical and Pharmaceutical Technologies (#2020-27).

### Collection of human endometrial tissues

Endometrial biopsy samples of mid-secretory phase (18–22 days of natural cycles) were collected from repeated implantation failure (RIF) patients that pregnancy failed after two consecutive transplants of high-quality blastocysts (*n* = 16) and control women (*n* = 7) at the Reproductive Medicine Center of the First Affiliated Hospital of Wenzhou Medical University. The control group had no previous history of adverse pregnancy and received assisted reproductive treatment due to male factors, and the pregnancy was successful. The tissue samples were stored in liquid nitrogen for 24 h and then transferred to –80 °C for long-term storage. The sample collection for this study was approved by the Medical Ethics Committees of Shanghai Institute for Biomedical and Pharmaceutical Technologies (Ref # PJ 2020-13) and the Reproductive Medicine Center of the First Affiliated Hospital of Wenzhou Medical University (Ref # 2019-07). All samples were collected after informed consent was obtained. No significant differences in the average age or gestational week at sampling were observed between the RIF patients and the control women (Supplementary Table S1).

### Fluorescent in situ hybridization assay of miR-3074-5p

Paraffin-embedded sections of uterine tissues collected from GD7.5 pregnant 3074-KI and WT mice were cut at 5μm thick, mounted on positively charged slides (Fisher Scientific, PA, United States). MiRNA in situ hybridization was performed. Briefly, tissue sections were baked at 60 °C for 20 min and soaked in 2 changes of Dewaxing Transparent Liquid, 15 min each. Dehydrate in 2 changes of pure ethanol for 5 min each. Then, followed respectively by dehydrating in gradient ethanol of 85%and 75% ethanol 5 min each. Soak in DEPC dilution (Sangon Biotech, Shanghai, China). The slides are boiled in the retrieval solution for 10–15 min and naturally cooled. Mark the objective tissue with liquid blocker pen, according to the characteristics of tissues. Proteinase K (20 μg/ml) (Ambion, United States) working solution was add to cover objectives and incubate at 37°C for 10 min. Wash in pure water, then wash three times in PBS (pH7.4) on a Rocker device, 5 min each. Endogenous peroxidases were blocked in 1% hydrogen peroxide (Sigma-Aldrich). Pre-hybridization solution was added to each section and incubate for 1 h at 37 °C. Remove the pre-hybridization solution, add the Cy3-modified probe corresponding to mature miR-3074-5p (sequence: 5ʹ-CTGGCTCAGTTCAGCAGGAAC-3ʹ) hybridization solution with concentration of 40 nM, and incubate the section in a humidity chamber and hybridize 2 h at 55°C. Remove the hybridization solution. Wash sections in 2×SSC for 10 min at 37 °C. To wash sections twice in 1×SSC for 5 min each time at 37 °C, and to wash in 0.5 × SSC for 10 min at room temperature. Formamide washing solution can be added if there are more non-specific hybrids. Sections were incubated with DAPI for 8 min in the dark, and then mounting.

### Opal immunohistochemical (IHC) assay of CD86, CD206 and F4/80

Formalin-fixed paraffin-embedded tissue sections of uterine tissues collected from GD7.5 pregnant 3074-KI and WT mice were cut at 5μm thickness and mounted on slides. The tissue was deparaffinized and antigen retrieval in citrate buffer prior to multiple labeling with the OpalTM 7-Color Manual IHC Kit (Perkinelmer, MA, United States). The tissue slides were immersed twice in xylene for 10 min per immersion, then, they were successively soaked in 100% alcohol for 5 min, 85% alcohol for 5 min, 70% alcohol for 5 min and finally rinsed with distilled water. These slides were placed in the repair solution and repaired with low-fire microwave (20 min for the first time and 10 min for each subsequent time). Slides were rinsed with TBST for 5 min, incubated in 5% bovine serum albumin for 10 min at room temperature (RT), and incubated with anti-CD206 primary antibody (Abcam, Cambridge, United Kingdom) for 30 min at RT followed by washing with TBST (twice, 5 min each). Slides were incubated with secondary antibody supplied within IHC kit for 10 min at RT, washed with TBST (twice, 5 min each), incubated with the Opal 620 fluorophore working solution (dilution: 1:100) for 10 min at RT, and washed with TBST (twice, 5 min each). After cooling, the process was repeated for four additional rounds for labeling with anti-CD86 (Cell Signaling Technology, MA, United States), and F4/80 (Abcam) followed by secondary labeling with the respective fluorophore working solution. Cell nuclei were labeled with DAPI for 5 min at RT, washed with TBST for 5 min, and coverslips were mounted with Antifade Mounting Medium. For multispectral image capture and analysis, the slides were scanned using the Vectra Ploris System (Perkinelmer). The chromogenic IHC-stained slides were scanned by using the bright field protocol, and the fluorescent uniplex and multiplex IHC staining were imaged using the fluorescence protocol ranging from 480 to 780 nm, to extract fluorescent intensity information from the images. A similar approach was used to build the spectral library using the InForm 2.6.0 image analysis software (Perkinelmer).

### Enzyme-linked immunosorbent assay (ELISA) of IL-1β and TNF-α

ELISA was applied to evaluate the levels of IL-1β and TNF-α in mouse uterine tissue lysate. For tissue total protein extraction, 30 mg of uterine tissue collected from GD8.5 pregnant mice was excised, and transferred into a microcentrifuge tube. 300 μl of lysis buffer (20 mM Tris pH 7.4, 250 mM NaCl, 1.5% Triton X-100, 2 mM EDTA, 1 mM PMSF, containing protease inhibitor cocktail) was added, followed by homogenate using a mini-homogenizer. Supernatant of tissue lysate was spin at 12,000 × *g* for 15 min at 4 °C. Then, the total protein samples were extracted and quantified using the Bradford method (Bio-Rad, United States). The ELISA for IL-1β and TNFα was accomplished following the instruction of manufacture (Elabscience Biotechnology Co., Wuhan, China). Briefly, prepare all reagents, working standards, and samples. Remove excess microplate strips from the plate frame, return them to the foil pouch containing the desiccant pack, reseal and return to 4 °C storage. Add 100 μl of all sample or standard to appropriate wells. Add 100 μl of the diluted Biotinylated Detection Ab to each well. Seal the plate and incubate for 1 h at 37 °C. Wash each well with 3 × 350 μl 1X Wash Buffer. Add 100 μl of the diluted HRP Conjugate to each well. Seal the plate and incubate for 30 min at 37 °C. Wash each well with 5 × 350 μl 1X Wash Buffer. Complete removal of liquid at each step is essential for good performance. After the last wash invert the plate and tap gently against clean paper towels to remove excess liquid. Add 100 μl of TMB Solution to each well and incubate for 10–15 min in the dark. Add 100 μl of Stop Solution to each well. Record the OD at 450 nm using Infinite 200 Pro M Plex (TECAN, Switzerland). A standard curve was run concurrently in every plate using dilution buffer provided by the manufacturer and the sample concentration was calculated based on the standard curve and dilution factor.

### Generation and treatment of human THP1-derived macrophages

THP1 cells were induced to differentiate into macrophages (THP1-derived Mϕs) as previously described. Briefly, THP1 cells were treated with 200 nM phorbol 12-myristate 13-acetate (PMA, Sigma, United States) followed by 24 h of incubation in RPMI-1640 medium to obtain a macrophage-like M0 state. Then, miR-3074-5p mimics (5′-GUUCCUGCUGAACUGAGCCAG-3′), siRNA specifically against ERα (ERα-siRNA:5′-UCCGAGUAUGAUCCUACCAGAdTdT-3′), or negative control sequence (NC:5′-UUCUCCGAACGUGUCACGUTT-3′), synthesized by Shanghai Gene Pharma Co., Ltd., were respectively transfected into THP1-derived Mϕs using Lipofectamine 2000 according to the manufacturer’s instructions (Invitrogen, Thermo Fisher Scientific, United States).

### Isolation and differentiation of mouse bone marrow-derived macrophages (BMDMs)

Primary BMDMs of C57BL/6 mice were prepared as previously described. In brief, bone marrow cells were aseptically collected from female mice aged 6-8 weeks (purchased from SIPPR/BK Laboratory Animal Company). The mice were sacrificed, and the collection area was sterilized with 75% ethanol. Then, cells were isolated by flushing the mouse leg bones with phosphate-buffered saline (PBS). The cells were incubated in red blood cell lysis buffer (Solarbio, Beijing, China) before centrifugation. The cells were then cultured for 7 days in DMEM containing 20% FBS, 0.055 mM 2-mercaptoethanol (Gibco), antibiotic-antimycotic (Gibco), and 30% conditioned medium from L929 cells expressing macrophage colony-stimulating factor (M-CSF). Nonadherent cells were removed.

### Dual-Luciferase reporter assays

To validate whether ERα was a direct target of miR-3074-5p, the 3ʹ-UTR sequence of the ERα mRNA was amplified from human genomic DNA. Both the wild-type and mutated 3ʹUTRs of ERα gene were subcloned into the pRL-TK plasmid vector (Genechem, Shanghai, China) after firefly Luciferase site. The constructed recombinant plasmids were confirmed by sequencing. HEK293 cells were plated in the 24-well plate (5 × 10^4^ cells per well). Twenty-four hours after seeding, firefly luciferase reporter constructs were transiently co-transfected with miR-3074-5p mimic or negative control and pRL-TK renilla luciferase plasmid (Genechem) per well, using lipofectamine 2000 (Invitrogen). Twenty-four hours after transfection, the ratio of firefly to renilla luciferase was measured using the Dual-Luciferase Reporter Assay System (Promega, Greek).

### Formation of trophoblast spheroids and in vitro implantation assay

The human first trimester extravillous trophoblast cell line, HTR-8/SVneo, was kindly provided by Dr Hongmei Wang, Institute of Zoology, Chinese Academy of Sciences, China, and the human endometrial stromal cell line, T-HESCs, was kindly provided by Professor Haibin Wang from the Medical College of Xiamen University, China. We used lentivirus to stably express pLVX-EGFP-IRES-puro vector in HTR-8/SVneo cells to make the cells express green fluorescent protein. PKH26 cell linker kit (MINI26-1KT; Sigma-Aldrich, United States) as used to label the membrane of HESC before co-culture. The co-culture system was applied to investigate the invasion of HTR-8/SVneo cell spheroids on decidualized T-HESC cells. Briefly, 1 ml culture medium containing 500,000 HTR-8/SVneo cells expressing EGFP (20 μl per drop) was plated onto the lid of 60 mm Petri dish (Corning Incorporated, United States) in regular arrays. The lid was inverted over the bottom of the PBS-filled Petri dish. The dish with the hanging drops were incubated at 37°C with 5% CO_2_ for 24 h to form cell spheres with a diameter of 150 nm. Subsequently, spheres were transferred to round-bottom glass shaker flasks containing 5 ml of complete medium and cultured on the shaker (130 rpm/min) at 37 °C with 5% CO_2_ for 48 h to form 300 nm spheroids. To recreate the structure of the endometrium, T-HESCs were seeded into 35 mm glass bottom dish (Corning Incorporated) at a density of 3 × 10^5^ cells per dish for 24 h. The in vitro decidualization of T-HESC were formed as described previously. Briefly, 0.5 mM Dibutyryl-cAMP (cAMP; MCE, NJ, United States), 10 nM β-estradiol (E2; Sigma-Aldrich), and 1 mM medroxyprogesterone 17-acetate (MPA; Sigma-Aldrich) in phenol red-free DMEM/F-12 containing 2% charcoal-stripped fetal bovine serum (Biological Industries, Israel) were applied for the in vitro decidualization of T-HESCs. PKH26 cell linker kit (MINI26-1KT; Sigma-Aldrich) was used to label the membrane of decidualized T-HESCs before co-culture. HTR8/SVneo spheroids were transferred into the decidualized T-HESCs. The outgrowth of spheroids was photographed by Nikon A1R Confocal real-time imaging of live cells for 10 h, and the outgrowth area of HTR-8/SVneo spheroids was calculated by NIS-Elements software (Nikon Corporation, Japan).

### Real-time quantitative PCR (RT-qPCR) assay

Total RNA was extracted with TRIzol Reagent (Invitrogen) and then reverse transcribed into cDNA (TaKaRa Bio, Inc. Japan) according to the manufacturer’s instructions. The synthesized cDNA was amplified with specific primers (Supplementary Table S2) and SYBR Green (TaKaRa Bio, Inc.) using a LightCycler 480 II real-time fluorescence quantitative PCR system (Roche, Basel, Switzerland). Triplicate samples were examined for each condition. The relative mRNA expression level was calculated using the 2 − ΔΔCt method.

### Flow cytometry assay

For antibody staining, THP1 cells were treated as indicated, washed, and incubated with CD80-PE and CD11b-FITC antibodies (BioLegend, United States) on ice for 30 min. Flow cytometric analysis was performed on a BD LSRFortessa system (BD Biosciences, United States), and the data were analyzed with FlowJo version 7.6.1 software. Serum cytokine concentration was measured using LEGENDplex Mouse inflammation Panel (#740446, BioLegend). The assay was performed according to the manufacturer’s instructions, and all samples were run in duplicate, and the data were analyzed with Qognit software, online software provided by BioLegend.

### Immunofluorescence staining

THP1 cells were differentiated as mentioned above and placed on coverslips (Fisher Scientific). The cells were fixed using 4% PFA, followed by blocking and permeabilization with 0.1% Igepal (Sigma–Aldrich, Inc.) in DPBS with 2% BSA (Amresco, OH, United States). Primary antibodies diluted in DPBS with 2% BSA (Sangon Biotech, Shanghai, China) were applied overnight at 4 °C. The cells were subsequently washed four times with DPBS before being incubated with the appropriate secondary antibodies (Invitrogen) diluted in 2% BSA in DPBS. The coverslips were washed four times with DPBS before being mounted on slides using mounting solution (Thermo Fisher Scientific). Confocal images were acquired with a Nikon A1R confocal system.

### Immunoprecipitation

The 293FT cells were plated in 6-well plates, cultured overnight and transfected with plasmids by lipofectamine2000 according to the manufacturer’s instructions. Specifically, the cells were co-transfected by pcDNA3.1-His-vector+pEGFP-ASC+pCMV-flag-vector, pcDNA3.1-His-vector+pEGFP-ASC+pCMV-flag-NLRP3 and pcDNA3.1-His-ERα + pEGFP -ASC+pCMV-flag-NLRP3, respectively. Twenty-four hours after transfection, cells were harvested in cold phosphate-buffered saline (PBS) and washed with PBS. Then, the cell pellets were suspended in lysis buffer (50 mM Tris-HCl, 120 mM NaCl, 1% Triton X-100., and protease inhibitor) and incubated on ice for 30 min. After centrifugation of the cell lysates (15 min, 12,000 × *g*, 4 °C), supernatant samples were used for IP. Anti-flag agarose resin (10 μl) (Yeasen; #20585ES08) was incubated with protein lysates (1 mg of protein) at 4 °C overnight with agitation. The beads were retained after three washes with lysis buffer. Next, 1× loading buffer was added to dissociate immunoprecipitates, and Western blotting was performed accordingly.

### Protein extraction and western blot analysis

For preparation of whole-cell extracts, cells were washed with ice-cold PBS, incubated with RIPA lysis buffer (Sangon Biotech) containing 1 mM PMSF and protease inhibitor cocktail (Selleck, Shanghai, China) on ice, and then homogenized with an ultrasonic cell disruptor. The supernatant of the cell and tissue lysates was centrifuged at 12,000 × g for 15 min at 4 °C. Cell nuclear protein was extracted using a Nucleoprotein Extraction Kit (Sangon Biotech) according to the manufacturer’s instructions. Approximately 50 μg of total protein from each sample was subjected to SDS-PAGE, and the separated proteins were transferred to nitrocellulose membranes (Merck Millipore, Darmstadt, Germany). Blots were incubated with the appropriate primary antibodies diluted in TBST (containing 0.1% Tween 20 and 2% BSA) for 1 h at room temperature. Then, the blots were washed and incubated with appropriate secondary antibodies and detected using an Odyssey CLx Imaging System (LI-COR, United States).

### Statistical analysis

At least three biological replicates were performed for all experiments unless otherwise indicated. A two-tailed Student’s *t* test was used for statistical analyses of paired observations. Differences between means were accepted as statistically significant at the 95% level (*p* < 0.05). One-way ANOVA was applied for comparisons between multiple groups as appropriate.

### Supplementary information

Supplementary materials

original blots

Supplementary movie 1

Supplementary movie 2

Supplementary movie 3

Supplementary movie 4

## Data Availability

All data generated or analyzed during this study are included in this published article and its supplementary information files.
