# Supplementary material for: Increased miR-3074-5p expression promotes M1 polarization and pyroptosis of macrophages via ERα/NLRP3 pathway and induces adverse pregnancy outcomes in mice
Source: Cell Death Discov. 2024 Apr 10;10:171. doi: 10.1038/s41420-024-01941-4 (PMC11006911; doi:10.1038/s41420-024-01941-4)
Supplement: Supplementary file 1 — Supplementary materials [file 41420_2024_1941_MOESM1_ESM.docx]

**
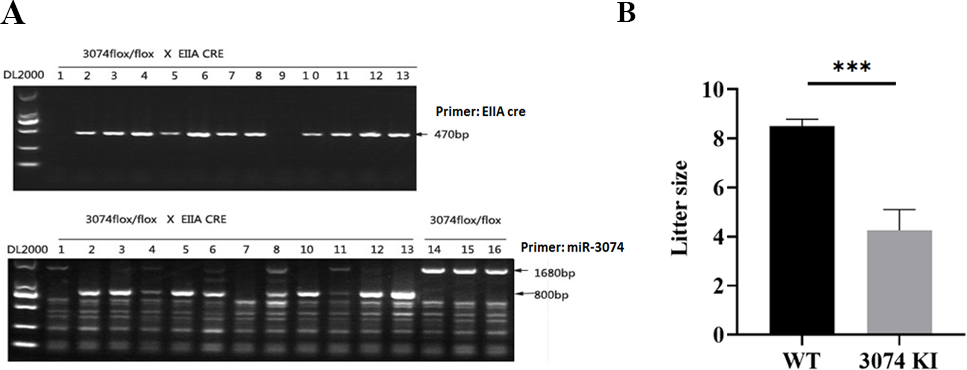
**

**Fig S1. Genotype identification of offsprings and the litter sizes of pregnant mice**

**A,** Genotype identification of miR-3074-5p knock-in (3074 KI) mice. Upper: PCR results using the primers of EIIA cre, and the PCR product was 470 bp. Below: PCR results using the primers of miR-3074-flox, and the PCR products were 1680 bp and 800 bp. 3074flox/flox × EIIA CRE: miR-3074-5p-flox/flox mice were mated with EIIA-cre mice; Offsprings No. 2, 3, 5, 10, 12 and 13 were identified as 3074 KI mice. **B,** Statistical results of litter sizes of wild-type (WT) pregnant mice (n=4) and 3074 KI pregnant mice (n=4). Data represented as mean ± SEM, ***: p<0.001.


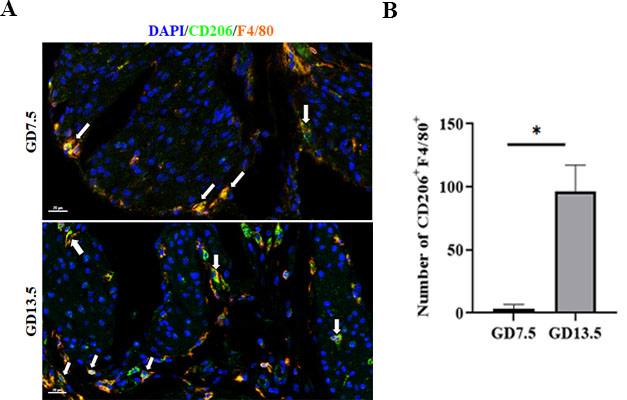


**Fig S2. The relative quantity of M2-type decidual macrophages in uterine tissues of pregnant mice**

**A,** The representative images of uterine tissues collected from wild-type pregnant mice at GD7.5 and GD13.5 (vaginal plug day was defined as GD0.5); M2-subtype (F4/80^+^/CD206^+^) decidual macrophages (dMφs) were stained in the paraffin sections, Orange: F4/80^+^, green: CD206^+^; Scale bar=20μm; **B,** the staining-positive cell number of M2-subtype (F4/80^+^/CD206^+^) dMφs in uterine tissues of pregnant mice at GD7.5 (n=3) and GD13.5(n=3); data are shown as the mean±SEM, *: p <0.05.

**
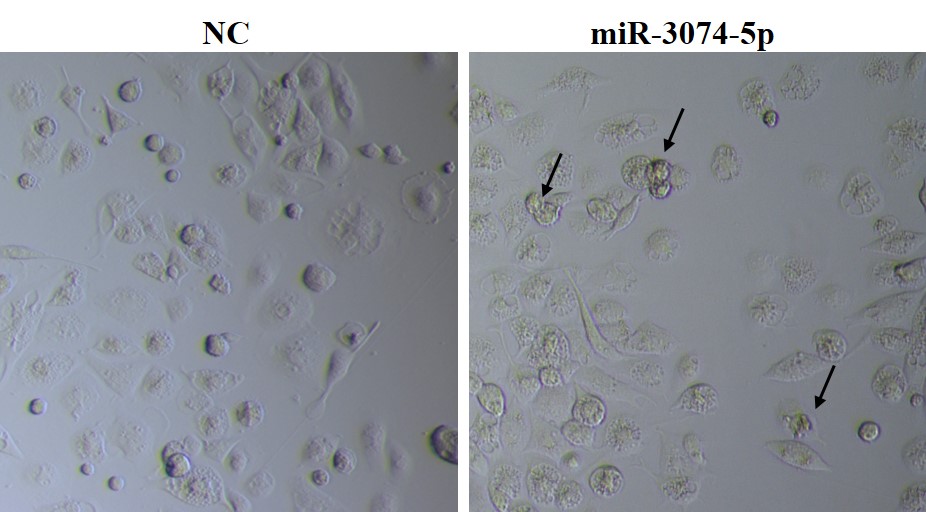
**

**Fig S3. The cell membrane damage phenotype of macrophages was observed under light microscopy**

The representative images of THP1-derived macrophages that respectively transfected with negative control fragment (NC) (left) and miR-3074-5p mimics (miR-3074-5p) (right). The black arrows in the image show cells with damaged cell membranes.


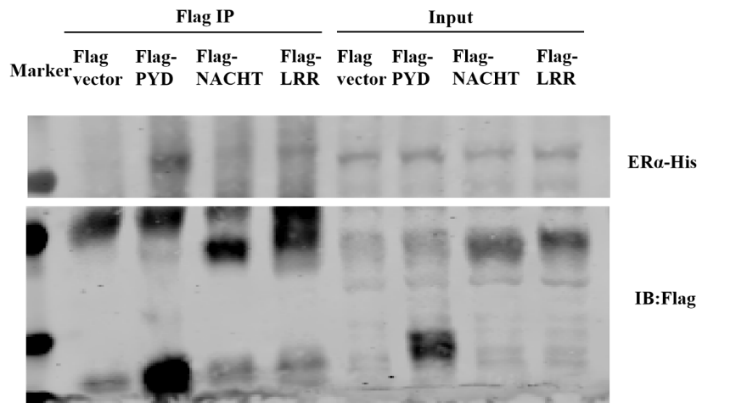


**Fig S4. NLRP3 interacted with ERα through the PYD domain**

Anti-Flag immunoprecipitation from lysates of 293T cells transfected with the indicated expression vectors. Immunoblots were analyzed with antibodies respectively against His (upper) and flag (below). Flag vector: cells transfected with vector plasmid expressed Flag protein; Flag-PYD: cells transfected with recombinant plasmid expressed Flag/NLRP3-PYD domain fusion protein; Flag-NACHT: cells transfected with recombinant plasmid expressed Flag/NLRP3-NACHT domain fusion protein; Flag-LRR: cells transfected with recombinant plasmid expressed Flag/ NLRP3-LRR fusion protein.


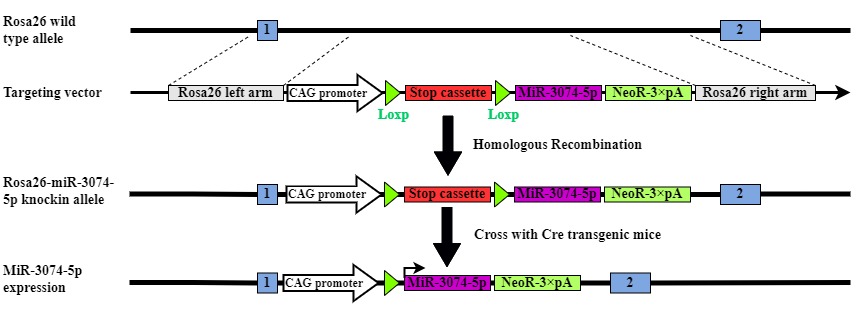


**Fig** **S5. Construction diagram of miR-3074-5p^flox/flox^ mice**

For the KI model, the "CAG promoter-Kozak-Mouse Fau CDS-WPRE-BGH pA" cassette will be cloned into intron 1 of ROSA26. To engineer the donor vector, homology arms will be generated by PCR using BAC clone as a template. Ribonucleoprotein (RNP) will be co-injected into fertilized eggs with the donor vector for mice production. The pups will be genotyped by PCR followed by sequencing analysis.

**Table S1.** Clinical characteristics of repeated implantation failure (RIF) patients and healthy control women (Control) whose endometrial biopsy were collected in this study

| **Control** | **RIF** |  |
| --- | --- | --- |
| **n=7** | **n=16** | **p value** |
| 30.29 ± 2.69 | 32.69 ± 3.63 | 0.133 |
| 0.00 ± 0.00 | 3.33 ± 0.72 | ＜0.001 |
| 0.57 ± 0.79 | 1.75 ± 1.16 | ＜0.05 |
| 0.14 ± 0.38 | 0.00 ± 0.00 | 0.147 |
| 0.29 ± 0.76 | 0.56 ± 0.74 | 0.366 |

**Table S2.** Primers used for real-time PCR

| **Primer name** | **Sequence (5′to3′)** |
| --- | --- |
| Homo CD80 Forward | GGCCCGAGTACAAGAACCG |
| Homo CD80 Reverse | TCGTATGTGCCCTCGTCAGAT |
| Homo actin Forward | CATGTACGTTGCTATCCAGGC |
| Homo actin Reverse | CTCCTTAATGTCACGCACGAT |
| Homo IL-6 Forward | ACTCACCTCTTCAGAACGAATTG |
| Homo IL-6 Reverse | CCATCTTTGGAAGGTTCAGGTTG |
| Homo TNF-α Forward | CCTCTCTCTAATCAGCCCTCTG |
| Homo TNF-α Reverse | GAGGACCTGGGAGTAGATGAG |
| Homo ERα Forward | CAAGTGGTTTCCTCGTGTCTAAAGC |
| Homo ERα Reverse | TGTTGAGTGTTGGTTGCGAGG |
| Mus TNF-α Forward | CCCTCACACTCAGATCATCTTCT |
| Mus TNF-α Reverse | GCTACGACGTGGGCTACAG |
| Mus IL-6 Forward | TAGTCCTTCCTACCCCAATTTCC |
| Mus IL-6 Reverse | TTGGTCCTTAGCCACTCCTTC |
| Mus iNOS Forward | GTTCTCAGCCCAACAATACAAGA |
| Mus iNOS Reverse | GTGGACGGGTCGATGTCAC |
| Mus GAPDH Forward | ACCCAGAAGACTGTGGATGG |
| Mus GAPDH Reverse | TTCAGCTCAGGGATGACCTT |

**TableS3.** Information of antibodies used in this study

| **Antibodies** | **Catalog Number** | **Company** |
| --- | --- | --- |
| Estrogen Receptor α (D8H8) Rabbit mAb | #8644 | Cell Signaling Technology |
| F4/80 (D2S9R) XP® Rabbit mAb | #70076 | Cell Signaling Technology |
| Anti-Mannose Receptor (CD206) antibody | ab8918 | abcam |
| Normal Rabbit IgG | #2729 | Cell Signaling Technology |
| CD86 (E5W6H) Rabbit mAb | #19589 | Cell Signaling Technology |
| Anti-IL-1 beta antibody | ab254360 | abcam |
| Anti-NLRP3 antibody | ab263899 | abcam |
| Anti-Caspase-1 antibody | ab207802 | abcam |
| Anti-NF-kB p65 antibody | ab32536 | abcam |
| Anti-NF-kB p65 (phospho S276) | Ab183559 | abcam |
| GSDMD Polyclonal antibody | 20770-1-AP | proteintech |
| IRDye 800CW Goat anti-Rabbit IgG (H + L) | 925-32211 | LI-COR |
| Donkey anti-rabbit (H+L) Cy3 | AP182C | EMD Millipore |
| Donkey anti-Mouse IgG (H+L) Highly Cross-Adsorbed Secondary Antibody, Alexa Fluor 488 | A-21202 | Thermo Fisher |
