# Supplementary material for: Increased miR-3074-5p expression promotes M1 polarization and pyroptosis of macrophages via ERα/NLRP3 pathway and induces adverse pregnancy outcomes in mice
Source: Cell Death Discov. 2024 Apr 10;10:171. doi: 10.1038/s41420-024-01941-4 (PMC11006911; doi:10.1038/s41420-024-01941-4)

**The blots marked by the red rectangle are the target proteins.**

**Figure 2E**

**ERα**


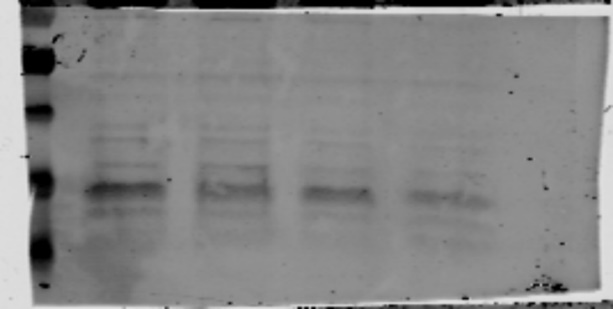


**β-actin**


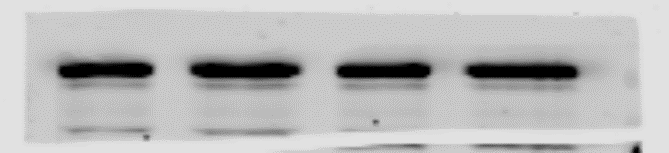


**Figure 3C**

**Pro-IL-1β**


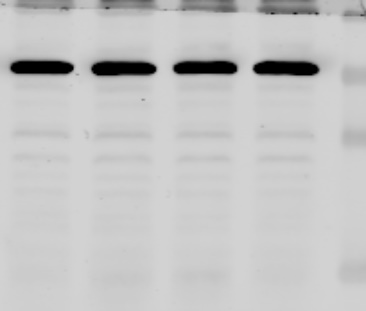


**Cleaved-IL-1β**


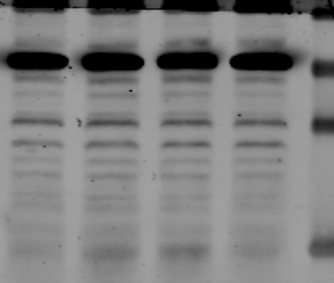


**β-actin**


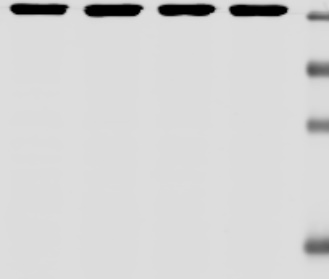


**Figure 3D**

**Pro-caspase1**


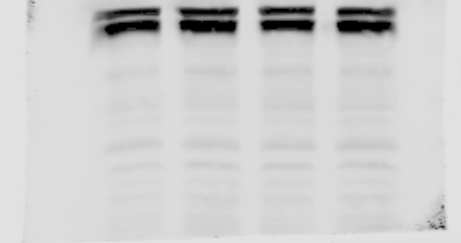


**Cleaved -caspase1**


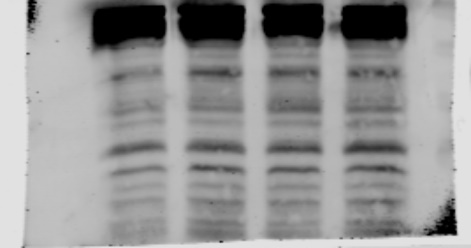


**β-actin**


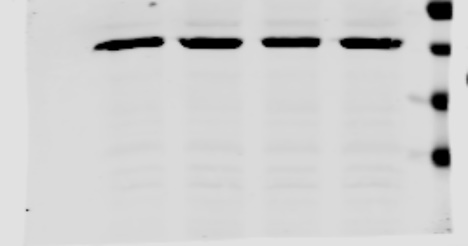


**Figure 3E**

**NLRP3**

**
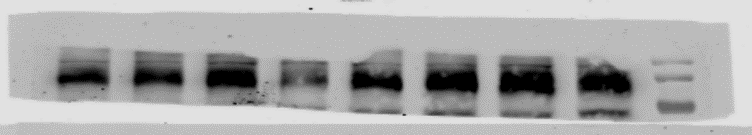
**

**GSDMD**


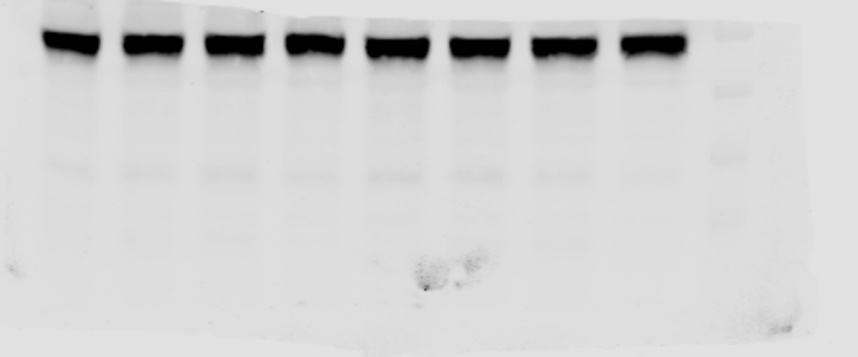


**Cleaved –GSDMD**

**
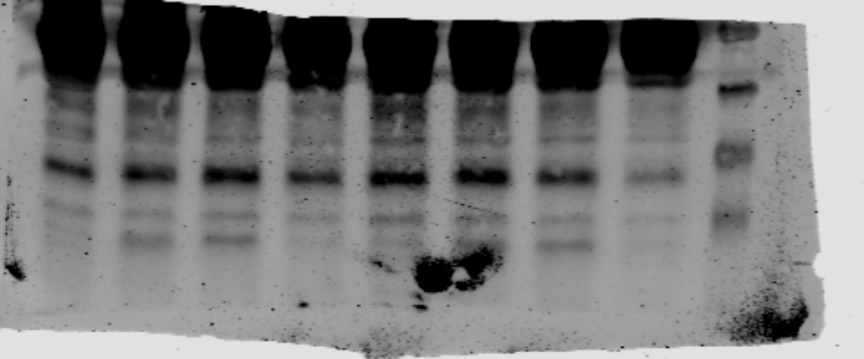
**

**actin**


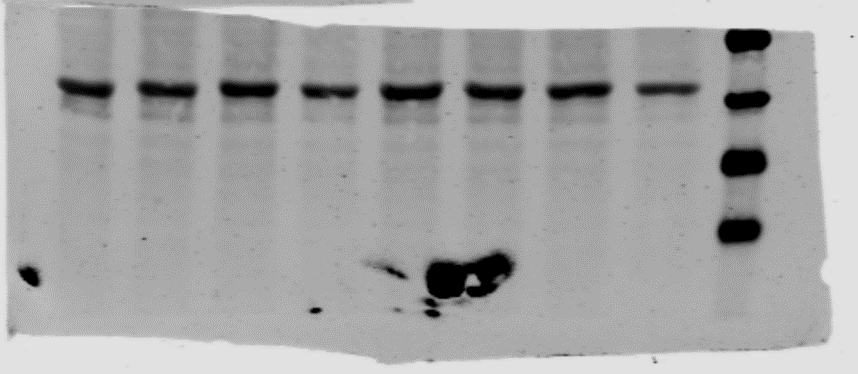


**Figure 3I**

**NLRP3**


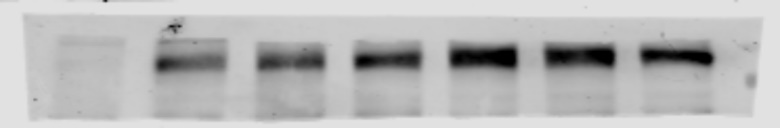


**GSDMD**


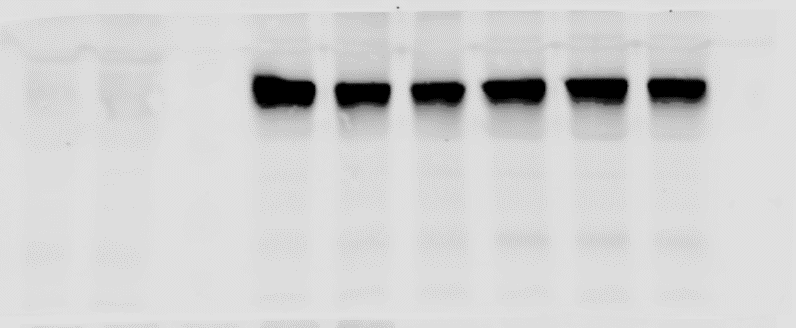


**Cleaved –GSDMD**


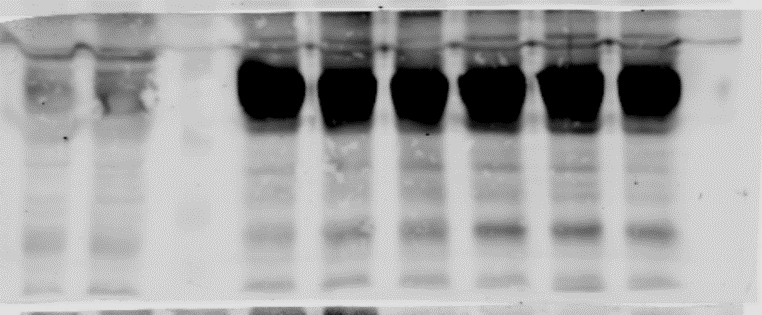


**actin**


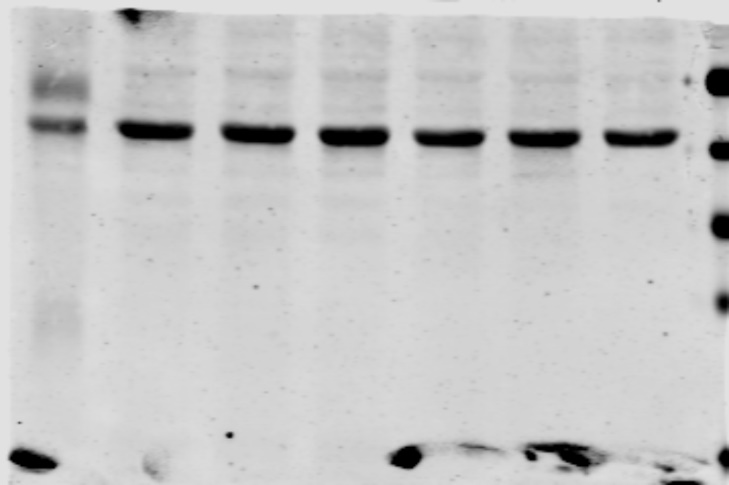


**Figure 4A**

**p-p65**


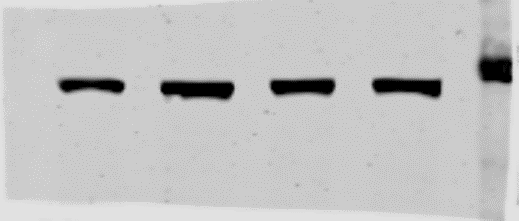


**actin**


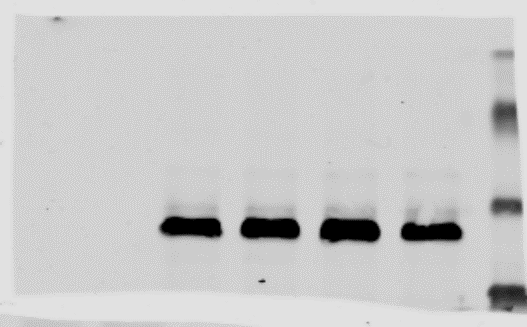


**Figure 4F**

**FLAG-NLRP3**


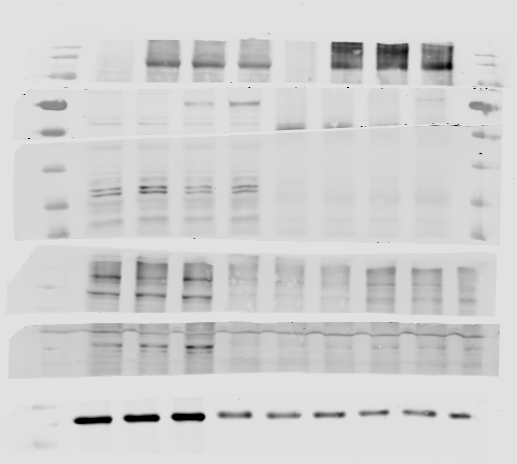


**ERα-His**


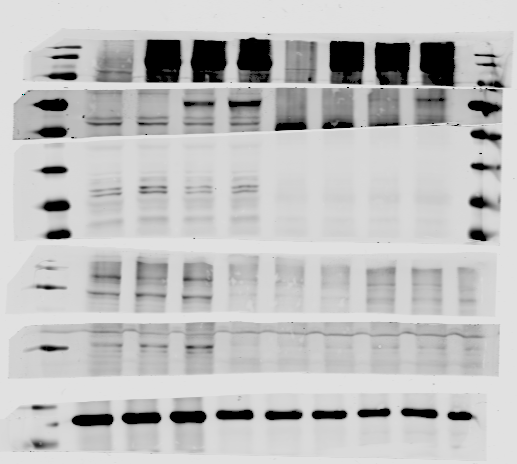


**Figure 4G**

**Flag-ERα**


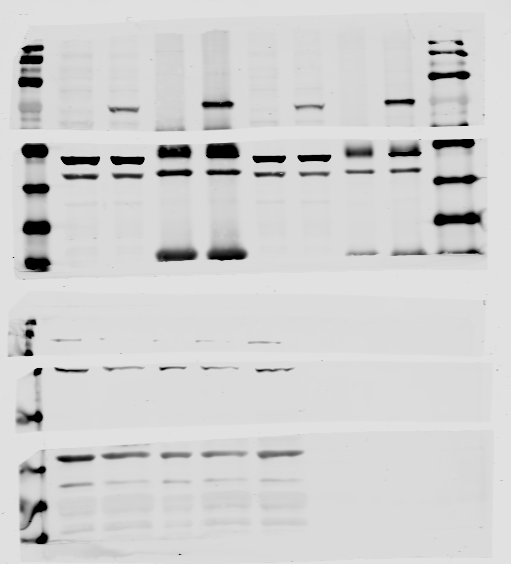


**ASC-GFP**


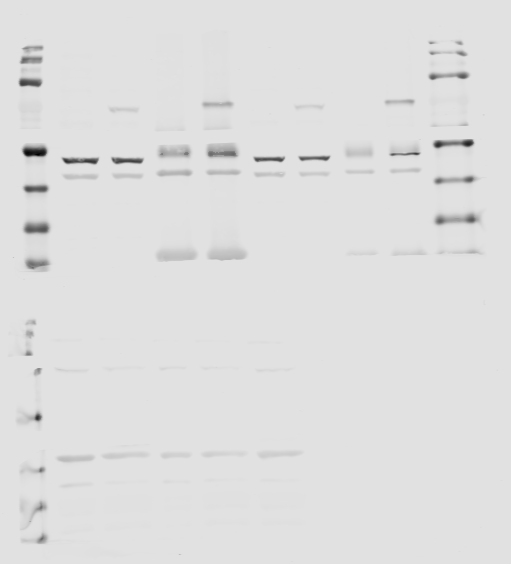


**Figure 4H**

**Flag-NLRP3**


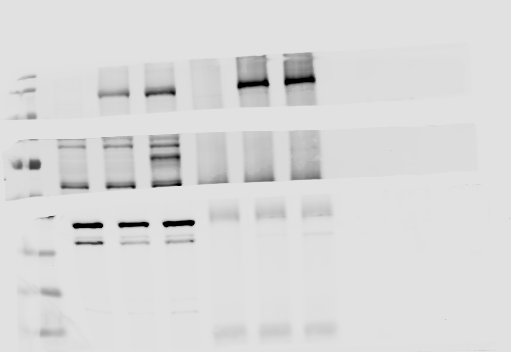


**ASC-GFP**


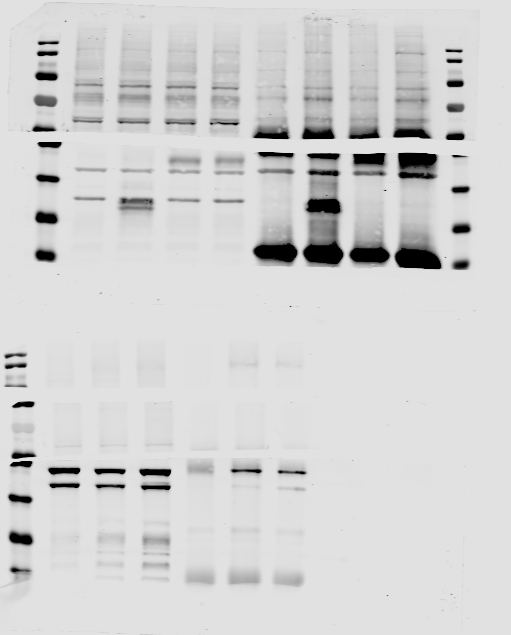


**Fig S4.**

**ERα-His**


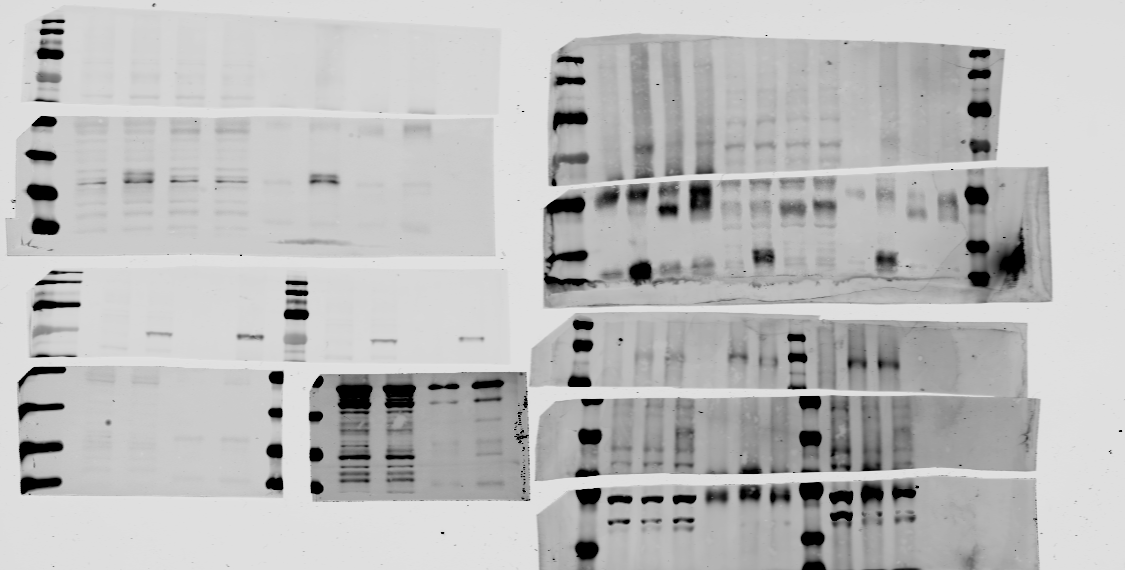


**Flag**


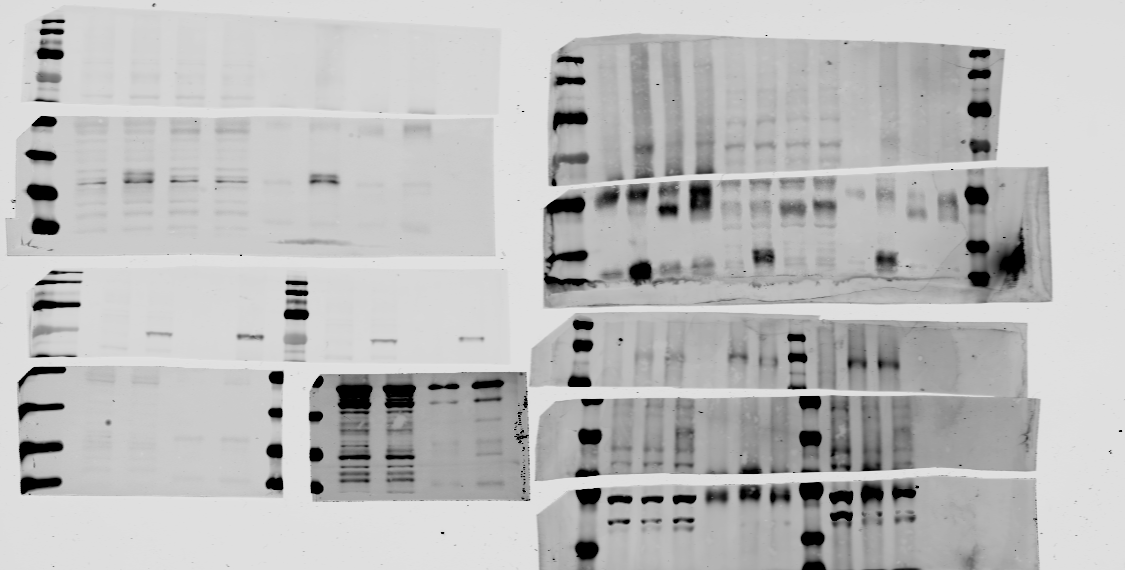

Supplement: Supplementary file 2 — original blots [file 41420_2024_1941_MOESM2_ESM.docx]
